# Clinical factors associated with carbapenem resistance and in-hospital mortality in *Klebsiella pneumoniae* bloodstream infection: a 7-year retrospective cohort study

**DOI:** 10.3389/fmicb.2026.1872672

**Published:** 2026-10-08

**Authors:** Na Xu, Yi Liang, Wen Zhu, Feng Lin, Yang Zhang, Jia Li

**Affiliations:** 1 Medical Laboratory, Shidong Hospital Affiliated to University of Shanghai for Science and Technology, Shanghai, China; 2 Department of Hospital Infection Control, Shidong Hospital Affiliated to University of Shanghai for Science and Technology, Shanghai, China

**Keywords:** in-hospital mortality, antimicrobial susceptibility, carbapenem resistance, *Klebsiella pneumoniae*, bloodstream infection

## Abstract

**Background:**

Carbapenem-resistant *Klebsiella pneumoniae* bloodstream infection (CRKP BSI) is associated with substantial mortality and limited therapeutic options, yet institutional epidemiology and risk profiles vary. We evaluated antimicrobial susceptibility, non-blood-site detection, factors associated with carbapenem resistance, and in-hospital all-cause mortality among patients with *K. pneumoniae* BSI at a secondary-care hospital over seven years.

**Methods:**

We retrospectively analyzed 141 adults with *K. pneumoniae* BSI hospitalized at Shidong Hospital, Shanghai, China, from January 2019 through December 2025, including 49 patients with CRKP BSI and 92 with carbapenem-susceptible *K. pneumoniae* BSI (CSKP BSI). Clinical and microbiological data were analyzed using univariable comparisons and multivariable logistic regression.

**Results:**

Crude in-hospital mortality was higher among patients with CRKP BSI than among those with CSKP BSI (40.8% vs. 16.3%; *p* = 0.002). Among the tested CRKP isolates, high *in vitro* susceptibility to ceftazidime/avibactam and tigecycline was observed. In multivariable analysis, pulmonary disease (aOR, 9.441; 95% CI, 2.958–30.136; *p* < 0.001), urinary catheterization before BSI onset (aOR, 13.821; 95% CI, 3.987–47.904; *p* < 0.001), prior carbapenem exposure (aOR, 3.982; 95% CI, 1.274–12.448; *p* = 0.018), and prior cephalosporin exposure (aOR, 11.341; 95% CI, 3.411–37.713; *p* < 0.001) were associated with carbapenem resistance. Non-blood-site detection of *K. pneumoniae* was more frequent in CRKP BSI than in CSKP BSI (73.5% vs. 35.9%; *p* < 0.001). In the overall mortality model, sepsis or septic shock assessed at BSI onset or within 48 h thereafter was associated with higher odds of in-hospital mortality (aOR, 4.738; 95% CI, 1.916–11.720; *p* < 0.001), whereas the adjusted association of CRKP BSI status with mortality did not reach statistical significance.

**Conclusion:**

In multivariable analysis, pulmonary disease, urinary catheterization before BSI onset, prior carbapenem exposure, and prior cephalosporin exposure were associated with carbapenem resistance. Although crude mortality was higher among patients with CRKP BSI, sepsis or septic shock was associated with higher odds of mortality after adjustment for age and CRKP BSI status, whereas the adjusted association of CRKP BSI status with mortality did not reach statistical significance. These findings support targeted infection prevention, antimicrobial stewardship, appropriate urinary catheter use, and early recognition and management of sepsis.

## Introduction

*Klebsiella pneumoniae* is a major opportunistic pathogen in healthcare settings and a leading cause of Gram-negative bacterial bloodstream infection (BSI), second only to *Escherichia coli* (Hafiz et al., 2023; Cheng et al., 2024). Carbapenems, known for their broad-spectrum activity and potent bactericidal effects, have long been the cornerstone of treatment for moderate-to-severe infections caused by multidrug-resistant Enterobacterales. However, with their widespread global use, the detection rate of carbapenem-resistant strains has continued to rise. Carbapenem-resistant *Klebsiella pneumoniae* (CRKP) has emerged as one of the most concerning resistant pathogens in healthcare-associated infection control and clinical antimicrobial therapy (Li Y. et al., 2020; Cao et al., 2022).

Data from the China Antimicrobial Surveillance Network (CHINET, http://www.chinets.com/) indicate that the resistance rate of clinical *K. pneumoniae* isolates to imipenem increased from 3% in 2005 to 24.8% in 2023, with a concurrent increase in meropenem resistance from 2.9 to 26.0%. In the cohort reported by Hovan et al., *K. pneumoniae* was the most frequently identified pathogen, accounting for 47.3% (69/146) of carbapenem-resistant Enterobacterales BSI cases (Hovan et al., 2021). Bloodstream infections caused by CRKP significantly impact patient morbidity and mortality (Falcone et al., 2023; Alicino et al., 2015), posing a major global public health challenge in antimicrobial therapy. Multiple studies have confirmed that carbapenem resistance is a risk factor for increased mortality among patients with *K. pneumoniae* BSI (Cheng et al., 2024; Chen J. et al., 2022). Consequently, the high mortality associated with CRKP BSI, coupled with limited therapeutic options, has emerged as a critical issue requiring urgent resolution in both healthcare-associated infection control and clinical antimicrobial management.

Numerous studies have investigated risk factors for CRKP infection, implicating prior antimicrobial exposure, invasive procedures, immunocompromised status, and intensive care unit (ICU) admission as potential contributors to CRKP BSI. However, study conclusions vary across regions and institutions, and discrepancies persist regarding the independent risk factors for CRKP BSI (Li Y. et al., 2020; Cao et al., 2022; Zheng et al., 2017). To address this gap, we conducted a 7-year retrospective study of 141 hospitalized patients with *K. pneumoniae* BSI at our hospital (January 2019–December 2025), including 49 patients with CRKP BSI and 92 with carbapenem-susceptible *K. pneumoniae* (CSKP) BSI. We characterized temporal trends, antimicrobial resistance profiles, departmental distribution, detection of *K. pneumoniae* from non-blood sites, and local susceptibility patterns over the study period. Using univariable comparisons and multivariable logistic regression, we evaluated factors associated with carbapenem resistance among patients with *K. pneumoniae* BSI and in-hospital mortality. This study provides real-world epidemiological and clinical data to support early recognition of high-risk patients, targeted infection prevention and control strategies, and locally informed antimicrobial therapy.

## Materials and methods

### Study design and population

This retrospective study was conducted at Shidong Hospital, a secondary hospital in Shanghai, China. Patients with *K. pneumoniae* BSI were identified through the microbiology laboratory database between January 2019 and December 2025. All adult inpatients (≥18 years of age) with at least one blood culture positive for *K. pneumoniae* were included. For patients with multiple positive blood cultures during the study period, only the first episode was included. Exclusion criteria were as follows: pregnancy, hospitalization for less than 48 h, and discharge against medical advice or transfer to another facility. The final analysis included 141 patients with *K. pneumoniae* BSI, comprising 49 patients with CRKP BSI and 92 patients with CSKP BSI as the comparison group. This study was reviewed and approved by the Ethics Committee of Shidong Hospital. The Ethics Committee evaluated the research protocol and the application for exemption from informed consent and granted approval (approval letter: 2023-058-01). All procedures and methodologies strictly adhered to relevant ethical guidelines and regulations. The exemption of informed consent was justified based on the nature of the research, which posed minimal risk to participants and did not involve the collection or publication of personally identifiable information.

### Antimicrobial susceptibility testing

Blood cultures were processed on the BacT/ALERT 3D system, and isolates were identified by matrix-assisted laser desorption/ionization time-of-flight mass spectrometry (MALDI-TOF MS); both systems were from bioMérieux (Marcy-l’Étoile, France). Kirby–Bauer disk diffusion with Oxoid disks (Basingstoke, UK) was performed for ampicillin/sulbactam, cefazolin, cefuroxime, ceftriaxone, gentamicin, and ceftazidime/avibactam. Minimum inhibitory concentrations (MICs) of cefoperazone/sulbactam, piperacillin/tazobactam, ceftazidime, cefepime, aztreonam, imipenem, amikacin, tobramycin, ciprofloxacin, levofloxacin, trimethoprim/sulfamethoxazole, tigecycline, colistin, minocycline, and doxycycline were determined using the VITEK 2 Compact system with AST-N335 cards (bioMérieux). Where applicable, we interpreted susceptibility results using the Clinical and Laboratory Standards Institute (CLSI) M100 edition adopted for each testing year, with interpretive criteria updated annually (29th–35th editions for 2019–2025, respectively). Tigecycline results were interpreted using U.S. Food and Drug Administration (FDA) breakpoints for Enterobacterales. Colistin susceptibility categories were extracted from routine laboratory reports using European Committee on Antimicrobial Susceptibility Testing (EUCAST) v13.0 criteria for Enterobacterales (bracketed MIC breakpoints: S ≤ (2), R > (2) mg/L). These thresholds do not by themselves establish clinical susceptibility; confirmatory broth microdilution was not routinely performed. *Escherichia coli* ATCC 25922 and *Pseudomonas aeruginosa* ATCC 27853 served as quality-control strains.

### Definitions

BSI onset was defined as the time of collection of the first positive blood culture. ICU length of stay was defined as the duration of ICU care (in days) during the entire index hospitalization, including post-BSI ICU time. Hospital-acquired BSI was defined as BSI occurring ≥ 48 h after hospital admission. Sepsis or septic shock was identified from the documented clinical diagnosis in the medical record and assessed at BSI onset or within the first 48 h thereafter. These diagnoses were not systematically adjudicated by retrospective application of Sepsis-3 criteria.

CRKP was defined as resistance to at least one carbapenem according to CLSI interpretive criteria, based on an MIC of ≥4 mg/L for imipenem or meropenem or ≥2 mg/L for ertapenem. Multidrug resistance (MDR) was defined as acquired non-susceptibility to at least one agent in three or more antimicrobial categories. Conventional β-lactam/β-lactamase inhibitor combinations (BLBLIs) included piperacillin/tazobactam, cefoperazone/sulbactam, and ampicillin/sulbactam. Antibiotic exposure was defined as receipt of intravenous or oral antibiotics for >48 h during the period from 90 days to 48 h before BSI onset.

For patient-level non-blood-site analyses, positivity was defined as at least one non-blood specimen yielding *K. pneumoniae* during the index hospitalization. Timing was determined by the earliest positive non-blood-site culture and classified as before, on the same calendar day as, or after BSI onset. Single-site and multisite detection were defined as positivity at one site and at least two distinct non-blood sites, respectively. For site-specific analyses, the denominator was the number of patients sampled at the corresponding site, with each patient counted once per site.

### Data collection

Data were retrospectively collected from the hospital’s electronic medical record system and included the following categories: (1) Demographic characteristics: age, sex, and total length of hospital stay. (2) Comorbidities and underlying conditions: Hypertension, diabetes mellitus, pulmonary disease, cardiovascular disease, cerebrovascular disease, hepatic insufficiency, renal insufficiency, and solid tumors. (3) Invasive procedures: Urinary catheterization, arterial or venous catheterization, nasogastric tube placement, gastric tube placement, endotracheal intubation, invasive mechanical ventilation, blood purification, bronchoscopy, enema, surgical intervention, and puncture. Procedures initiated before and after BSI onset were recorded separately. (4) Hospitalization and ICU care: Hospitalization within the preceding three months, any ICU admission during the index hospitalization, BSI onset in the ICU, and ICU length of stay. (5) Disease severity: Acute Physiology and Chronic Health Evaluation II (APACHE II) score. (6) Special therapies: Administration of corticosteroids, chemotherapy/radiotherapy, or immunosuppressive agents. (7) Antimicrobial exposure and resistance: History of antibiotic exposure and antimicrobial susceptibility testing results. Therapeutic regimens were summarized by antimicrobial class over the entire index hospitalization for descriptive comparisons between hospital-acquired and non-hospital-acquired BSI. Patient-level concordance between empirical therapy and subsequent susceptibility results was not assessed. (8) Non-blood-site *K. pneumoniae* detection: Isolation of *K. pneumoniae* from body sites other than blood during the current hospitalization. A clinically adjudicated primary source of bacteremia was not available in the analytical dataset. Non-blood-site culture results were analyzed as microbiological detection variables and were not used to determine the primary infection source. (9) Clinical outcomes: Patient prognosis and mortality.

### Statistical analysis

Statistical analyses were conducted using WHONET 5.6, IBM SPSS Statistics version 27.0, and GraphPad Prism version 10.6.0. Continuous variables with a normal distribution were reported as mean ± standard deviation (SD), whereas non-normally distributed variables were reported as median and interquartile range (IQR). APACHE II scores were dichotomized at ≥ 20 for clinical interpretability in risk-factor analyses but were not included in the primary mortality model because they were available only for a subset of patients and would have substantially reduced the analyzable sample. In univariable analyses, categorical variables were compared using the chi-square test or Fisher’s exact test, as appropriate, and continuous variables were compared using Student’s t-test or the Mann–Whitney U test. All tests were two-tailed, and *p* < 0.05 was considered statistically significant. Site-specific analyses in Supplementary Table S3 were exploratory, and no adjustment was made for multiple comparisons.

Multivariable logistic regression was used to examine factors associated with carbapenem resistance and mortality. Pulmonary disease, invasive procedures, and antibiotic exposure were considered *a priori* clinically relevant, but the final covariate sets were not fully prespecified. Variables with univariable *p* < 0.05 were considered for multivariable modeling, and eligibility and final selection were evaluated during data analysis using clinical relevance, biological plausibility, measurement timing, exposure frequency, and potential conceptual overlap. Variable-specific inclusion and exclusion decisions are detailed in Supplementary Table S5.

Total hospital length of stay was excluded from the CRKP-versus-CSKP model because it included post-BSI time. The variable “any ICU admission during the index hospitalization” was excluded from both models because it did not distinguish between ICU admissions before and after BSI onset. Tables 1, 2 report ICU length of stay as a descriptive clinical-course variable, with exploratory group comparisons. Because this measure included post-BSI time and could reflect the consequences of BSI and subsequent illness severity, it was not interpreted as a strictly pre-BSI exposure or a baseline prognostic factor. It was therefore not included in either reported final multivariable model.

**Table 1:** Clinical characteristics of CRKP BSI vs. CSKP BSI patients, 2019–2025.

Items	CSKP (*n* = 92)	CRKP (*n* = 49)	*p* value
Male	65 (70.7%)	33 (67.3%)	0.704
Age	69 (63.3–74.8)	73 (64.5–85.0)	**0.028**
Total length of stay, days	17 (12.0–26.8)	27 (17.0–49.0)	**<0.001**
Time from admission to BSI onset, days	4 (1–10.8)	12 (5–25.5)	**<0.001**
Post-BSI length of stay, days	11.5 (7–15.8)	12 (6–20.5)	0.550
Hypertension	51 (55.4%)	32 (65.3%)	0.285
Diabetes	44 (47.8%)	24 (49.0%)	>0.05
Pulmonary disease	21 (22.8%)	31 (63.3%)	**<0.001**
Respiratory failure	6 (6.5%)	6 (12.2%)	0.342
Cardiovascular disease	24 (26.1%)	18 (36.7%)	0.246
Cerebrovascular disease	19 (20.7%)	15 (30.6%)	0.217
Hepatic insufficiency	16 (17.4%)	3 (6.1%)	0.073
Renal insufficiency	18 (19.6%)	14 (28.6%)	0.291
Solid tumor	39 (42.4%)	10 (20.4%)	**0.010**
Malignant hematological disease	4 (4.3%)	5 (10.2%)	0.276
Digestive system disease	18 (19.6%)	7 (14.3%)	0.495
Urinary system disease	4 (4.3%)	1 (2.0%)	0.658
Sepsis or septic shock	14 (15.2%)	23 (46.9%)	**<0.001**
Hospitalization within 3 months	36 (39.1%)	32 (65.3%)	**0.004**
APACHE II ≥ 20	8 (53.3%)	17 (60.7%)	0.750
Any ICU admission during the index hospitalization	17 (18.5%)	27 (55.1%)	**<0.001**
BSI onset in the ICU	8 (8.7%)	23 (46.9%)	**<0.001**
ICU length of stay during the index hospitalization, days	15 (8–24)	22 (14–34)	**0.025**
Chemotherapy or radiotherapy before BSI onset	14 (15.2%)	7 (14.3%)	>0.05
Use of immunosuppressants before BSI onset	1 (1.1%)	1 (2.0%)	>0.05
Urinary catheterization before BSI onset	9 (9.8%)	29 (59.2%)	**<0.001**
Nasogastric tube placement before BSI onset	18 (19.6%)	28 (57.1%)	**<0.001**
Gastric tube placement before BSI onset	2 (2.2%)	3 (6.1%)	0.342
Arterial or venous catheterization before BSI onset	16 (17.4%)	25 (51.0%)	**<0.001**
Blood purification before BSI onset	6 (6.5%)	13 (26.5%)	**0.002**
Invasive respiratory support before BSI onset	14 (15.2%)	21 (42.9%)	**<0.001**
Bronchoscopy before BSI onset	5 (5.4%)	8 (16.3%)	0.062
Enema before BSI onset	4 (4.3%)	11 (22.4%)	**0.003**
Open surgery within 3 months before BSI onset	4 (4.3%)	11 (22.4%)	**0.003**
Non-open surgery within 3 months before BSI onset	18 (19.6%)	7 (14.3%)	0.495
Drainage before BSI onset	6 (6.5%)	5 (10.2%)	0.514
Puncture before BSI onset	2 (2.2%)	5 (10.2%)	0.050
Invasive procedures ≥ 3 before BSI onset	15 (16.3%)	30 (61.2%)	**<0.001**
Any antibiotic exposure	27 (29.3%)	44 (89.8%)	**<0.001**
Carbapenem exposure	9 (9.8%)	23 (46.9%)	**<0.001**
Conventional BLBLIs exposure	4 (4.3%)	13 (26.5%)	**<0.001**
Cephalosporin exposure	12 (13.0%)	28 (57.1%)	**<0.001**
Quinolones exposure	4 (4.3%)	13 (26.5%)	**<0.001**
Aminoglycosides exposure	1 (1.1%)	1 (2.0%)	>0.05
Linezolid exposure	1 (1.1%)	2 (4.1%)	0.277
Glycopeptides exposure	3 (3.3%)	7 (14.3%)	**0.033**
Tigecycline exposure	0	4 (8.2%)	**0.013**
Polymyxin exposure	1 (1.1%)	1 (2.0%)	>0.05
Non-blood-site *K. pneumoniae* detection	33 (35.9%)	36 (73.5%)	**<0.001**

Continuous variables are presented as median (interquartile range), and categorical variables as *n* (%). *p* values <0.05 are bolded. APACHE II percentages were calculated among patients with available scores (CSKP, *n* = 15; CRKP, *n* = 28). ICU length of stay covered the entire index hospitalization, including post-BSI time, and was summarized among patients with any ICU admission during that hospitalization (CSKP, *n* = 17; CRKP, *n* = 27). The ICU-length-of-stay comparison was descriptive and exploratory and was not interpreted as evidence of a pre-BSI risk factor or a causal effect. ICU-length-of-stay data were complete for these 44 patients; all remaining variables except APACHE II were complete for the full cohort. The borderline comparison for puncture before BSI onset involved only seven exposed patients and should be interpreted cautiously. CRKP, carbapenem-resistant *Klebsiella pneumoniae*; CSKP, carbapenem-susceptible *Klebsiella pneumoniae*; ICU, intensive care unit; BLBLIs, β-lactam/β-lactamase inhibitor combinations; BSI, bloodstream infection. APACHE II, Acute Physiology and Chronic Health Evaluation II.

**Table 2:** Univariable analysis of factors associated with in-hospital all-cause mortality in the overall *K. pneumoniae* BSI cohort and resistance-phenotype subgroups.

Items	KP BSI			CRKP BSI			CSKP BSI		
	Survival (*n* = 106)	Death (*n* = 35)	*p* value	Survival (*n* = 29)	Death (*n* = 20)	*p* value	Survival (*n* = 77)	Death (*n* = 15)	*p* value
Male	75 (70.8%)	23 (65.7%)	0.672	21 (72.4%)	12 (60.0%)	0.536	54 (70.1%)	11 (73.3%)	>0.05
Age, years	69 (63–78)	73 (69–79)	**0.006**	69 (61.5–84)	78.5 (69.5–85)	**0.039**	69 (63–76)	69 (69–73)	0.261
Total length of hospital stay, days	17.5 (12–30)	24 (15–43)	0.070	28 (17–52)	25 (15.8–47.8)	0.736	16 (10–25.5)	20 (15–32)	0.133
Time from admission to BSI onset, days	5 (1–12.5)	14 (4–22)	**0.005**	12 (4–24)	17.5 (6.3–26)	0.698	2 (0–10)	10 (4–16)	**0.015**
Post-BSI length of stay, days	12 (7–18)	11 (6–15)	0.436	14 (5–25)	10 (6–17.3)	0.562	12 (7–16.5)	11 (5–15)	0.524
Hypertension	60 (56.6%)	23 (65.7%)	0.429	17 (58.6%)	15 (75.0%)	0.361	43 (55.8%)	8 (53.3%)	>0.05
Diabetes	47 (44.3%)	21 (60.0%)	0.122	12 (41.4%)	12 (60.0%)	0.252	35 (45.5%)	9 (60.0%)	0.399
Pulmonary disease	35 (33.0%)	17 (48.6%)	0.110	20 (69.0%)	11 (55.0%)	0.375	15 (19.5%)	6 (40.0%)	0.099
Respiratory failure	7 (6.6%)	5 (14.3%)	0.172	3 (10.3%)	3 (15.0%)	0.677	4 (5.2%)	2 (13.3%)	0.252
Cardiovascular disease	34 (32.1%)	8 (22.9%)	0.395	13 (44.8%)	5 (25.0%)	0.230	21 (27.3%)	3 (20.0%)	0.751
Cerebrovascular disease	24 (22.6%)	10 (28.6%)	0.499	8 (27.6%)	7 (35.0%)	0.754	16 (20.8%)	3 (20.0%)	>0.05
Hepatic insufficiency	17 (16.0%)	2 (5.7%)	0.158	2 (6.9%)	1 (5.0%)	>0.05	15 (19.5%)	1 (6.7%)	0.455
Renal insufficiency	24 (22.6%)	8 (22.9%)	>0.05	9 (31.0%)	5 (25.0%)	0.754	15 (19.5%)	3 (20.0%)	>0.05
Solid tumor	35 (33.0%)	14 (40.0%)	0.540	5 (17.2%)	5 (25.0%)	0.720	30 (39.0%)	9 (60.0%)	0.159
Malignant hematological disease	5 (4.7%)	4 (11.4%)	0.226	3 (10.3%)	2 (10.0%)	>0.05	2 (2.6%)	2 (13.3%)	0.123
Digestive system disease	20 (18.9%)	5 (14.3%)	0.619	5 (17.2%)	2 (10.0%)	0.685	15 (19.5%)	3 (20.0%)	>0.05
Urinary system disease	3 (2.8%)	2 (5.7%)	0.598	0	1 (5.0%)	0.408	3 (3.9%)	1 (6.7%)	0.516
Sepsis or septic shock	18 (17.0%)	19 (54.3%)	**<0.001**	9 (31.0%)	14 (70.0%)	**0.010**	9 (11.7%)	5 (33.3%)	**0.048**
Hospitalization within 3 months	50 (47.2%)	18 (51.4%)	0.700	23 (79.3%)	9 (45.0%)	**0.017**	27 (35.1%)	9 (60.0%)	0.087
Any ICU admission during the index hospitalization	26 (24.5%)	18 (51.4%)	**0.006**	13 (44.8%)	14 (70.0%)	0.143	13 (16.9%)	4 (26.7%)	0.466
BSI onset in the ICU	15 (14.2%)	16 (45.7%)	**<0.001**	9 (31.0%)	14 (70.0%)	**0.010**	6 (7.8%)	2 (13.3%)	0.612
ICU length of stay during the index hospitalization, days	17.5 (8–30.3)	21 (14.3–38)	0.206	25 (12.5–33.5)	21.5 (15–38)	0.784	14 (5.5–24)	16 (9.8–60.5)	0.492
Chemotherapy or radiotherapy before BSI onset	15 (14.2%)	6 (17.1%)	0.784	4 (13.8%)	3 (15.0%)	>0.05	11 (14.3%)	3 (20.0%)	0.694
Use of immunosuppressants before BSI onset	1 (0.9%)	1 (2.9%)	0.436	1 (3.4%)	0	>0.05	0	1 (6.7%)	0.163
Urinary catheterization before BSI onset	25 (23.6%)	13 (37.1%)	0.129	17 (58.6%)	12 (60.0%)	>0.05	8 (10.4%)	1 (6.7%)	>0.05
Nasogastric tube placement before BSI onset	32 (30.2%)	14 (40.0%)	0.304	16 (55.2%)	12 (60.0%)	0.777	16 (20.8%)	2 (13.3%)	0.727
Gastric tube before BSI onset	4 (3.8%)	1 (2.9%)	>0.05	2 (6.9%)	1 (5.0%)	>0.05	2 (2.6%)	0	>0.05
Arterial or venous catheterization before BSI onset	24 (22.6%)	17 (48.6%)	**0.005**	13 (44.8%)	12 (60.0%)	0.387	11 (14.3%)	5 (33.3%)	0.128
Blood purification before BSI onset	15 (14.2%)	4 (11.4%)	0.783	11 (37.9%)	2 (10.0%)	**0.047**	4 (5.2%)	2 (13.3%)	0.252
Invasive respiratory support before BSI onset	23 (21.7%)	12 (34.3%)	0.175	10 (34.5%)	11 (55.0%)	0.240	13 (16.9%)	1 (6.7%)	0.452
Fiberoptic bronchoscopy before BSI onset	7 (6.6%)	6 (17.1%)	0.088	4 (13.8%)	4 (20.0%)	0.700	3 (3.9%)	2 (13.3%)	0.185
Enema before BSI onset	8 (7.5%)	7 (20.0%)	0.055	5 (17.2%)	6 (30.0%)	0.320	3 (3.9%)	1 (6.7%)	0.516
Open surgery within 3 months before BSI onset	13 (12.3%)	2 (5.7%)	0.358	9 (31.0%)	2 (10.0%)	0.162	4 (5.2%)	0	>0.05
Non-open surgery within 3 months before BSI onset	20 (18.9%)	5 (14.3%)	0.619	4 (13.8%)	3 (15.0%)	>0.05	16 (20.8%)	2 (13.3%)	0.727
Drainage before BSI onset	6 (5.7%)	5 (14.3%)	0.141	3 (10.3%)	2 (10.0%)	>0.05	3 (3.9%)	3 (20.0%)	0.053
Puncture before BSI onset	3 (2.8%)	4 (11.4%)	0.064	3 (10.3%)	2 (10.0%)	>0.05	0	2 (13.3%)	**0.025**
Invasive procedures ≥ 3 before BSI onset	29 (27.4%)	16 (45.7%)	0.059	17 (58.6%)	13 (65.0%)	0.769	12 (15.6%)	3 (20.0%)	0.706
Urinary catheterization after BSI onset	23 (21.7%)	14 (40.0%)	**0.045**	9 (31.0%)	9 (45.0%)	0.375	14 (18.2%)	5 (33.3%)	0.292
Nasogastric tube placement after BSI onset	17 (16.0%)	16 (45.7%)	**<0.001**	8 (27.6%)	11 (55.0%)	0.075	9 (11.7%)	5 (33.3%)	**0.048**
Gastric tube after BSI onset	0	1 (2.9%)	0.248	0	0	>0.05	0	1 (6.7%)	0.163
Arterial or venous catheterization after BSI onset	17 (16.0%)	18 (51.4%)	**<0.001**	7 (24.1%)	15 (75.0%)	**0.001**	10 (13.0%)	3 (20.0%)	0.439
Blood purification after BSI onset	4 (3.8%)	8 (22.9%)	**0.002**	2 (6.9%)	6 (30.0%)	0.050	2 (2.6%)	2 (13.3%)	0.123
Invasive respiratory support after BSI onset	11 (10.4%)	8 (22.9%)	0.085	2 (6.9%)	5 (25.0%)	0.105	9 (11.7%)	3 (20.0%)	0.406
Fiberoptic bronchoscopy after BSI onset	5 (4.7%)	4 (11.4%)	0.226	2 (6.9%)	3 (15.0%)	0.387	3 (3.9%)	1 (6.7%)	0.516
Enema after BSI onset	10 (9.4%)	7 (20.0%)	0.132	3 (10.3%)	6 (30.0%)	0.133	7 (9.1%)	1 (6.7%)	>0.05
Open surgery within 3 months after BSI onset	3 (2.8%)	0	0.574	1 (3.4%)	0	>0.05	2 (2.6%)	0	>0.05
Non-open surgery within 3 months after BSI onset	5 (4.7%)	1 (2.9%)	>0.05	1 (3.4%)	0	>0.05	4 (5.2%)	1 (6.7%)	>0.05
Drainage after BSI onset	7 (6.6%)	2 (5.7%)	>0.05	3 (10.3%)	1 (5.0%)	0.636	4 (5.2%)	1 (6.7%)	>0.05
Puncture after BSI onset	3 (2.8%)	2 (5.7%)	0.598	0	1 (5.0%)	0.408	3 (3.9%)	1 (6.7%)	0.516
≥ 3 types of invasive procedures after BSI onset	14 (13.2%)	16 (45.7%)	**<0.001**	4 (13.8%)	12 (60.0%)	**0.001**	10 (13.0%)	4 (26.7%)	0.234
Any antibiotic exposure	50 (47.2%)	21 (60.0%)	0.242	27 (93.1%)	17 (85%)	0.387	23 (29.9%)	4 (26.7%)	>0.05
Carbapenem exposure	16 (15.1%)	16 (45.7%)	**<0.001**	10 (34.5%)	13 (65.0%)	**0.046**	6 (7.8%)	3 (20.0%)	0.160
Conventional BLBLIs exposure	13 (12.3%)	4 (11.4%)	>0.05	10 (34.5%)	3 (15.0%)	0.191	3 (3.9%)	1 (6.7%)	0.516
Cephalosporin exposure	27 (25.5%)	13 (37.1%)	0.200	16 (55.2%)	12 (60.0%)	0.777	11 (14.3%)	1 (6.7%)	0.683
Quinolones exposure	9 (8.5%)	8 (22.9%)	**0.035**	6 (20.7%)	7 (35.0%)	0.331	3 (3.9%)	1 (6.7%)	0.516
Aminoglycosides exposure	0	2 (5.7%)	0.060	0	1 (5.0%)	0.408	0	1 (6.7%)	0.163
Linezolid exposure	2 (1.9%)	1 (2.9%)	>0.05	1 (3.4%)	1 (5.0%)	>0.05	1 (1.3%)	0	>0.05
Glycopeptides exposure	7 (6.6%)	3 (8.6%)	0.709	5 (17.2%)	2 (10.0%)	0.685	2 (2.6%)	1 (6.7%)	0.418
Tigecycline exposure	3 (2.8%)	1 (2.9%)	>0.05	3 (10.3%)	1 (5.0%)	0.636	0	0	>0.05
Polymyxin exposure	0	2 (5.7%)	0.060	0	1 (5.0%)	0.408	0	1 (6.7%)	0.163
Ceftazidime/Avibactam exposure	4 (3.8%)	0	0.572	4 (13.8%)	0	0.135	0	0	>0.05
Non-blood-site *K. pneumoniae* detection	51 (48.1%)	18 (51.4%)	0.846	21 (72.4%)	15 (75.0%)	1.000	30 (39.0%)	3 (20.0%)	0.241
CRKP BSI (vs. CSKP BSI)	29 (27.4%)	20 (57.1%)	**0.002**						

Continuous variables are presented as median (interquartile range), and categorical variables as *n* (%). *p* values <0.05 are bolded. The primary endpoint was in-hospital all-cause mortality. ICU length of stay covered the entire index hospitalization, including post-BSI time, and was summarized among patients with any ICU admission during that hospitalization (survivors/non-survivors: overall, *n* = 26/18; CRKP, *n* = 13/14; CSKP, *n* = 13/4). Post-BSI procedures and ICU length of stay were considered clinical-course variables; the corresponding comparisons were descriptive and exploratory and were not interpreted as evidence of baseline prognostic value or causal effects. The timing of ICU discharge or death may influence the observed duration. KP, *Klebsiella pneumoniae*; BSI, bloodstream infection; CRKP, carbapenem-resistant *Klebsiella pneumoniae*; CSKP, carbapenem-susceptible *Klebsiella pneumoniae*; ICU, intensive care unit; BLBLIs, β-lactam/β-lactamase inhibitor combinations.

Procedures initiated after BSI onset were excluded from the mortality model because they were considered clinical-course variables rather than baseline prognostic factors. Given the 35 in-hospital deaths, the primary mortality model included three clinically selected covariates: age, carbapenem-resistance phenotype (CRKP vs. CSKP), and sepsis or septic shock; the latter was assessed at BSI onset or within 48 h thereafter and served as an indicator of early illness severity.

Multicollinearity among retained covariates was assessed using tolerance and variance inflation factor (VIF) values, with VIF < 5 indicating no substantial multicollinearity. Results are reported as adjusted odds ratios (aORs) with 95% confidence intervals (CIs). The Hosmer–Lemeshow test was used to assess model goodness of fit. To assess sensitivity to potential small-sample and sparse-data bias, we refitted the CRKP-versus-CSKP model using Firth’s penalized logistic regression with the logistf package (version 1.26.1) in R (version 4.5.1), retaining the same 141 patients and four covariates. We calculated 95% penalized profile-likelihood confidence intervals and obtained *p*-values from penalized likelihood-ratio tests.

## Results

### Annual distribution and mortality of *Klebsiella pneumoniae* BSI, 2019–2025

A total of 141 patients with *K. pneumoniae* BSI were enrolled in this study, including 92 (65.2%) with CSKP BSI and 49 (34.8%) with CRKP BSI. The annual numbers of included *K. pneumoniae* BSI cases were 20 in 2019, 15 in 2020, 10 in 2021, 11 in 2022, 30 in 2023, 33 in 2024, and 22 in 2025. From 2019 to 2025, the annual proportion of patients with *K. pneumoniae* BSI among those with a positive blood culture ranged from 14.0 to 22.7%. The annual proportions of overall *K. pneumoniae* BSI, hospital-acquired *K. pneumoniae* BSI, and CRKP BSI fluctuated over the study period, with relatively lower proportions observed in the later years (Figure 1A).

**Figure 1:**
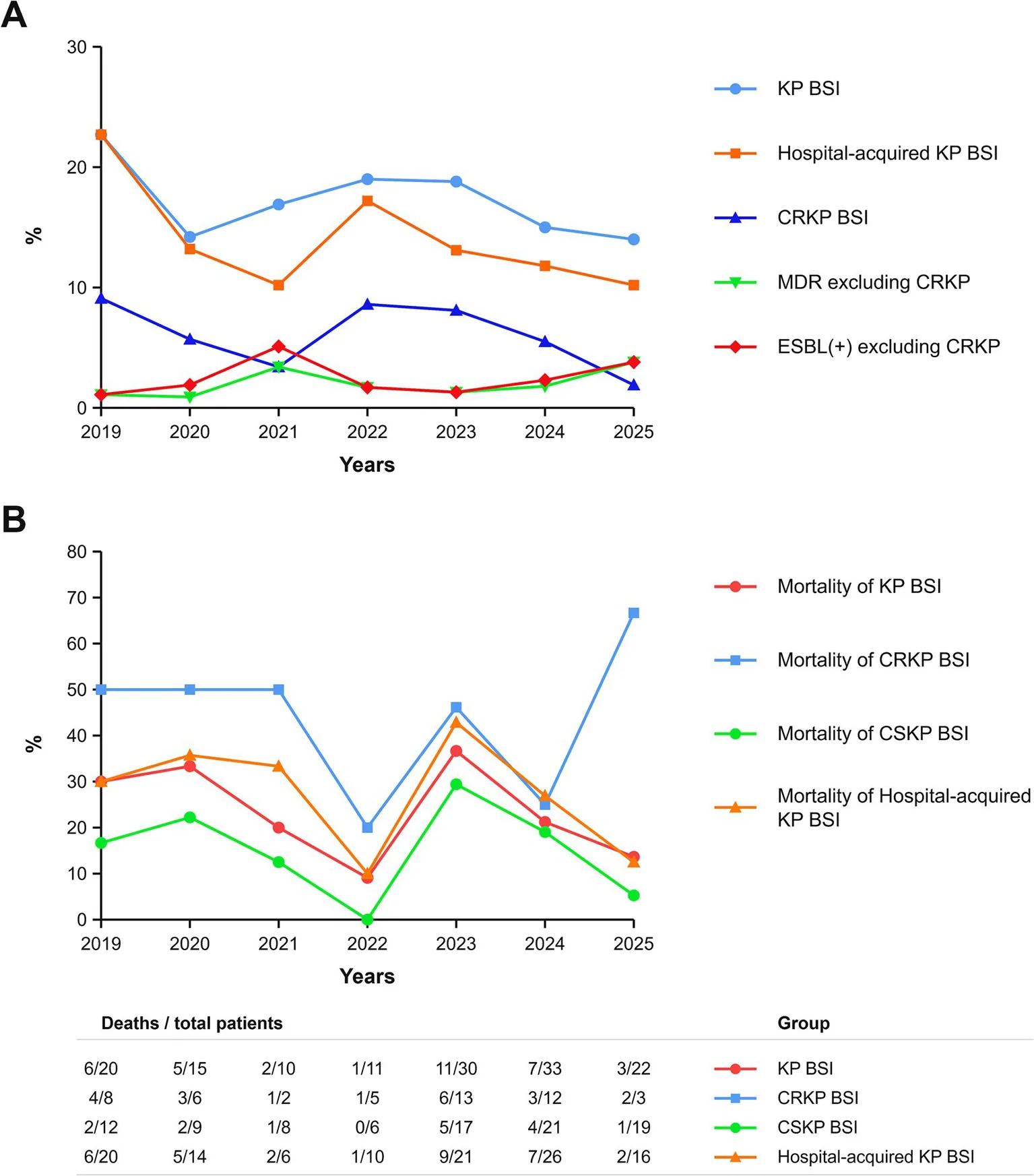
Annual trends (2019–2025) in **(A)** Proportions of *K. pneumoniae* BSI and hospital-acquired *K. pneumoniae* BSI by resistance phenotype, and **(B)** In-hospital all-cause mortality rates by *K. pneumoniae* resistance phenotype. In panel **A**, percentages were calculated using the number of patients with a positive blood culture in each year as the common denominator (*N* = 88, 106, 59, 58, 160, 220, and 157 for 2019–2025, respectively). Each patient was counted once within each calendar year. Values below panel **B** indicate deaths/total patients for each year and group. Annual mortality estimates should be interpreted descriptively because of the small number of cases in individual years. KP, *Klebsiella pneumoniae*; BSI, bloodstream infection; CRKP, carbapenem-resistant *Klebsiella pneumoniae*; CSKP, carbapenem-susceptible *Klebsiella pneumoniae;* MDR, multidrug-resistant; ESBL, extended-spectrum β-lactamase.

In-hospital mortality was significantly higher in the CRKP group (20/49, 40.8%) than in the CSKP group (15/92, 16.3%; *p* = 0.002). Overall mortality rates for *K. pneumoniae* BSI, CSKP BSI, and hospital-acquired *K. pneumoniae* BSI peaked in 2023 at 36.7, 29.4, and 42.9%, respectively. In 2025, the overall mortality rate for *K. pneumoniae* BSI decreased to 13.6%; however, the mortality rate for the CRKP subgroup reached its highest point during the study period at 66.7% (Figure 1B).

### Distribution by department at hospital admission

*K. pneumoniae* BSI was most frequently identified in patients from the general surgery department (21.3%), ICU (16.3%), emergency department (9.2%), oncology department (7.8%), and nephrology department (7.8%) (Figure 2A). Hospital-acquired *K. pneumoniae* BSI was most frequently identified in the surgery department (21.2%), the ICU (18.6%), and the nephrology department (8.8%), followed by the oncology, hematology, and interventional radiology departments (Figure 2B). CRKP BSI cases were most frequently identified in the ICU, followed by patients in the surgery and nephrology departments (Figure 2C).

**Figure 2:**
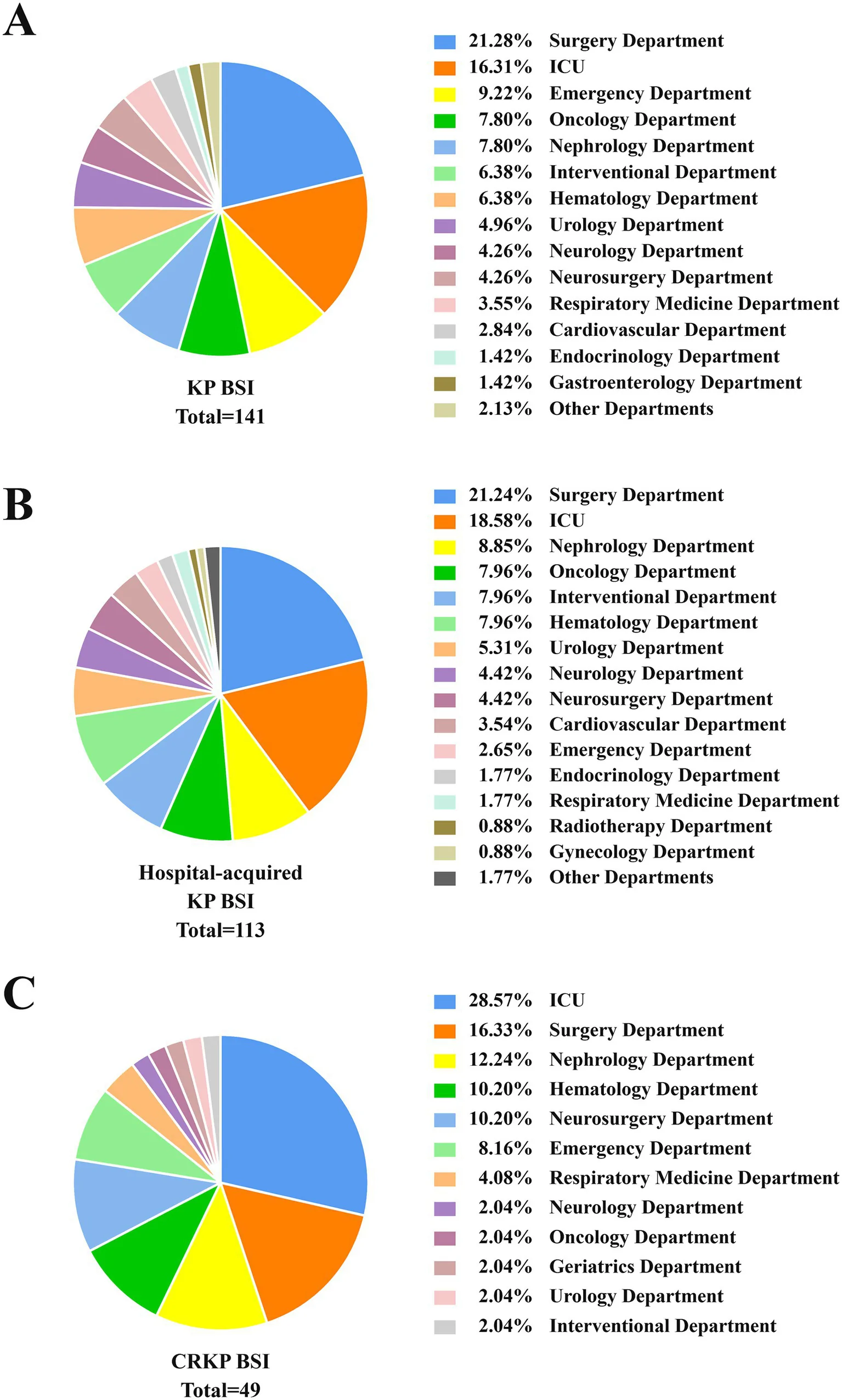
Departmental distribution of **(A)** Total *K. pneumoniae* BSI, **(B)** Hospital-acquired *K. pneumoniae* BSI, and **(C)** CRKP BSI, 2019–2025. Department was defined at hospital admission. KP, *Klebsiella pneumoniae*; CRKP, carbapenem-resistant *Klebsiella pneumoniae*; BSI, bloodstream infection; ICU, intensive care unit.

### Antimicrobial resistance in *Klebsiella pneumoniae* BSI

From 2019 to 2024, the percentage of *K. pneumoniae* BSI isolates resistant to imipenem ranged from 20.0 to 45.5%, then decreased to 13.6% in 2025 (Table 3).

**Table 3:** Annual antimicrobial resistance rates of *K. pneumoniae* isolates from BSI blood cultures, 2019–2025.

Antimicrobial agents	2019	2020	2021	2022	2023	2024	2025
	(*n* = 20)	(*n* = 15)	(*n* = 10)	(*n* = 11)	(*n* = 30)	(*n* = 33)	(*n* = 22)
Cefoperazone/Sulbactam	40.0	40.0	30.0	45.5	46.7	36.4	22.7
Ampicillin/Sulbactam	45.0	53.3	40.0	63.6	46.7	42.4	27.3
Piperacillin/Tazobactam	40.0	40.0	40.0	45.5	46.7	36.4	27.3
Cefazolin	50.0	53.3	50.0	63.6	50.0	42.4	36.4
Cefuroxime	45.0	46.7	50.0	63.6	50.0	42.4	36.4
Ceftazidime	45.0	46.7	40.0	54.5	46.7	39.4	36.4
Ceftriaxone	45.0	46.7	40.0	54.5	46.7	42.4	36.4
Cefepime	40.0	46.7	40.0	45.5	46.7	39.4	36.4
Aztreonam	40.0	40.0	20.0	45.5	43.3	36.4	13.6
Imipenem	40.0	40.0	20.0	45.5	43.3	36.4	13.6
Amikacin	15.0	20.0	20.0	27.3	43.3	24.2	13.6
Gentamicin	40.0	46.7	30.0	45.5	46.7	36.4	22.7
Tobramycin	45.0	46.7	30.0	45.5	43.3	36.4	22.7
Ciprofloxacin	60.0	60.0	40.0	54.5	46.7	42.4	36.4
Levofloxacin	65.0	60.0	40.0	45.5	46.7	39.4	36.4
TMP-SMZ	40.0	46.7	20.0	36.4	43.3	39.4	31.8
Tigecycline	10.0	6.7	10.0	9.1	6.7	9.1	4.5
Ceftazidime/Avibactam	-	-	-	-	0	0	4.5
Colistin	-	-	-	-	0	0	0
Minocycline	-	-	-	-	43.3	36.4	27.3
Doxycycline	-	-	-	-	43.3	36.4	31.8

Values are resistance percentages. For each agent tested in a given year, the denominator is the annual isolate count shown in the column heading. Colistin results reflect routine laboratory classifications; no confirmatory broth microdilution was performed. “-” indicates the antimicrobial agent was not tested in the corresponding year. TMP-SMZ, trimethoprim-sulfamethoxazole; BSI, bloodstream infection.

### Antimicrobial susceptibility of CRKP BSI isolates

Among the agents shown in Table 4, testing for ceftazidime/avibactam, colistin, minocycline, and doxycycline was limited to 2023–2025. Resistance rates among tested isolates were 61.2% (30/49) for amikacin, 71.4% (20/28) for doxycycline, 64.3% (18/28) for minocycline, and 69.4% (34/49) for trimethoprim–sulfamethoxazole. Susceptibility rates were 96.4% (27/28) for ceftazidime/avibactam and 95.9% (47/49) for tigecycline. All 28 CRKP isolates tested for colistin had VITEK 2-reported MICs ≤2 mg/L and were classified as susceptible in the original routine laboratory reports.

**Table 4:** Antimicrobial susceptibility profiles of CRKP bloodstream isolates for selected clinically relevant agents, 2019–2025.

Antimicrobial agents	Resistance, n/N (%)	Intermediate, n/N (%)	Susceptible, n/N (%)
Ceftazidime/Avibactam	1/28 (3.6)	0/28 (0.0)	27/28 (96.4)
Amikacin	30/49 (61.2)	0/49 (0.0)	19/49 (38.8)
Gentamicin	40/49 (81.6)	0/49 (0.0)	9/49 (18.4)
Tobramycin	41/49 (83.7)	0/49 (0.0)	8/49 (16.3)
TMP-SMZ	34/49 (69.4)	0/49 (0.0)	15/49 (30.6)
Colistin	0/28 (0.0)	0/28 (0.0)	28/28 (100.0)
Minocycline	18/28 (64.3)	4/28 (14.3)	6/28 (21.4)
Doxycycline	20/28 (71.4)	2/28 (7.1)	6/28 (21.4)
Tigecycline	2/49 (4.1)	0/49 (0.0)	47/49 (95.9)

Data are presented as n/N (%), where *n* is the number of isolates in each susceptibility category and *N* is the number tested for that agent. Results for ceftazidime/avibactam, colistin, minocycline, and doxycycline are limited to 2023–2025 and may not represent the full 2019–2025 CRKP cohort. Colistin categories reflect the original routine laboratory reports. No confirmatory broth microdilution was performed; these classifications do not represent reference-method-confirmed susceptibility. Percentages may not sum to 100% because of rounding. TMP-SMZ, trimethoprim-sulfamethoxazole; CRKP, carbapenem-resistant *Klebsiella pneumoniae*.

### Antibiotic exposure and utilization in hospital-acquired versus non-hospital-acquired *Klebsiella pneumoniae* BSI

Prior exposure to cephalosporins, carbapenems, and conventional BLBLIs was more common among patients with hospital-acquired than non-hospital-acquired *K. pneumoniae* BSI (all *p* < 0.05). For treatment, the proportions receiving cephalosporin- or carbapenem-containing regimens did not differ significantly between the groups (*p* = 0.211 and *p* = 0.295, respectively). A higher proportion of patients in the non-hospital-acquired group received conventional BLBLI-containing regimens (35.7% vs. 15.9%; *p* = 0.032) (Table 5).

**Table 5:** Prior antimicrobial exposure and antimicrobial use during hospitalization in patients with hospital-acquired and non-hospital-acquired *K. pneumoniae* BSI.

Antimicrobial agent or class	Non-hospital-acquired (*n* = 28)	Hospital-acquired (*n* = 113)	*p* value
Prior antimicrobial exposure			
Cephalosporins	3	37	**0.020**
Quinolones	1	16	0.194
Aminoglycosides	0	2	>0.05
Carbapenems	0	32	**<0.001**
Conventional BLBLIs	0	17	**0.024**
Polymyxins	0	2	>0.05
Glycopeptides	0	10	0.211
Linezolid	0	3	>0.05
Tigecycline	0	4	0.584
Ceftazidime/avibactam	0	4	0.584
Antifungal agents	0	9	0.205
Antimicrobial use during the index hospitalization			
Cephalosporins	17	53	0.211
Quinolones	12	29	0.102
Aminoglycosides	0	11	0.121
Carbapenems	11	58	0.295
Conventional BLBLIs	10	18	**0.032**
Polymyxins	0	10	0.211
Glycopeptides	3	17	0.765
Linezolid	1	8	0.688
Tigecycline	1	15	0.195
Ceftazidime/Avibactam	0	12	0.124
Antifungal agents	1	12	0.465

*p* values less than 0.05 are bolded. Conventional BLBLIs include piperacillin/tazobactam, cefoperazone/sulbactam, and ampicillin/sulbactam. Antimicrobial use refers to receipt of the listed agents at any time during the index hospitalization. Polymyxins included polymyxin B and colistin. BLBLIs, β-lactam/β-lactamase inhibitor combinations; BSI, bloodstream infection.

### Factors associated with carbapenem resistance among patients with *Klebsiella pneumoniae* BSI

#### Demographic characteristics

Patients in the CRKP BSI group had a significantly higher median age compared to those in the CSKP BSI group (73 years vs. 69 years; *p* = 0.028). The sex distribution did not differ significantly between the two groups. The CRKP BSI group had a significantly longer total length of hospital stay (median 27 days vs. 17 days; *p* < 0.001), as well as a significantly longer pre-BSI hospital stay (median 12 days vs. 4 days; *p* < 0.001). However, the two groups did not differ significantly in post-BSI hospital stay.

#### Underlying diseases and comorbidities

Subgroup analysis of underlying diseases and comorbidities showed that the prevalence of pulmonary disease was significantly higher in the CRKP BSI group than in the CSKP BSI group (63.3% vs. 22.8%; *p* < 0.001). Solid tumors were more common in the CSKP BSI group (42.4% vs. 20.4%; *p* = 0.01).

#### Disease severity and ICU admission

The proportion of patients developing sepsis or septic shock was significantly higher in the CRKP BSI group than in the CSKP BSI group (46.9% vs. 15.2%; *p* < 0.001). Furthermore, any ICU admission during the index hospitalization (55.1% vs. 18.5%; *p* < 0.001) and BSI onset in the ICU (46.9% vs. 8.7%; *p* < 0.001) were more frequent in the CRKP group than in the CSKP group. Among ICU-admitted patients, the CRKP BSI group had a significantly longer ICU length of stay during the index hospitalization than the CSKP BSI group (median 22 days vs. 15 days; *p* = 0.025).

#### Invasive procedures and antibiotic exposure

In univariable comparisons, pre-BSI urinary catheterization, nasogastric tube placement, arterial or venous catheterization, blood purification, invasive respiratory support, and enema, as well as open surgery within 3 months before BSI onset, were more frequent in the CRKP group than in the CSKP group. Prior exposure to any antibiotic, including carbapenems, conventional BLBLIs, cephalosporins, quinolones, glycopeptides, or tigecycline, was also more frequent in the CRKP group (all *p* < 0.05; Table 1).

#### Detection of *Klebsiella pneumoniae* at non-blood sites

During the index hospitalization, *K. pneumoniae* was detected at non-blood sites in 69 of 141 patients (48.9%), with a higher proportion in the CRKP group than in the CSKP group (73.5% vs. 35.9%; *p* < 0.001). Based on the earliest positive non-blood-site culture, detection before BSI onset was more frequent in the CRKP group (36.7% vs. 9.8%; *p* < 0.001), whereas same-day and post-BSI first detection did not differ significantly between groups (*p* = 0.484 and *p* = 0.429, respectively). Multisite detection was also more frequent in the CRKP group (28.6% vs. 6.5%; *p* < 0.001), whereas single-site detection did not differ significantly (Table 6). In exploratory analyses restricted to patients sampled at each site, sputum culture positivity was higher in the CRKP group (78.6% vs. 29.4%; *p* = 0.002), whereas no statistically significant differences were observed for bronchoalveolar lavage fluid, urine, or other non-blood sites (Supplementary Table S3).

**Table 6:** Patient-level non-blood-site *K. pneumoniae* detection according to bloodstream carbapenem-susceptibility phenotype.

Outcome	Overall (*n* = 141)	CRKP BSI (*n* = 49)	CSKP BSI (*n* = 92)	*p* value
A. Presence and timing of non-blood-site detection				
Any non-blood-site detection	69/141 (48.9%)	36/49 (73.5%)	33/92 (35.9%)	<0.001
Detection before or on the day of BSI onset	51/141 (36.2%)	28/49 (57.1%)	23/92 (25.0%)	<0.001
Before BSI onset	27/141 (19.1%)	18/49 (36.7%)	9/92 (9.8%)	<0.001
On the day of BSI onset	24/141 (17.0%)	10/49 (20.4%)	14/92 (15.2%)	0.484
After BSI onset	18/141 (12.8%)	8/49 (16.3%)	10/92 (10.9%)	0.429
B. Number of non-blood sites with detection				
Single-site detection	49/141 (34.8%)	22/49 (44.9%)	27/92 (29.3%)	0.094
Multisite detection	20/141 (14.2%)	14/49 (28.6%)	6/92 (6.5%)	<0.001

Values are presented as n/N (%). In Panels A and B, *N* denotes the total number of patients in each BSI group. Timing categories were mutually exclusive and determined by the earliest positive non-blood-site culture relative to BSI onset; “on the day of BSI onset” denotes the same calendar day as collection of the first positive blood culture. Multisite detection was defined as positive cultures from at least two distinct non-blood sites. The data do not distinguish colonization from infection or establish clonal relatedness between non-blood-site and bloodstream isolates. BSI, bloodstream infection; CRKP, carbapenem-resistant *Klebsiella pneumoniae*; CSKP, carbapenem-susceptible *Klebsiella pneumoniae*.

Multivariable logistic regression showed that pulmonary disease (aOR, 9.441; 95% CI, 2.958–30.136; *p* < 0.001), urinary catheterization before BSI onset (aOR, 13.821; 95% CI, 3.987–47.904; *p* < 0.001), carbapenem exposure (aOR, 3.982; 95% CI, 1.274–12.448; *p* = 0.018), and cephalosporin exposure (aOR, 11.341; 95% CI, 3.411–37.713; *p* < 0.001) were associated with CRKP BSI rather than CSKP BSI (Supplementary Table S1; Figure 3A). The Hosmer–Lemeshow test did not indicate a statistically significant lack of fit (*p* = 0.464).

**Figure 3:**
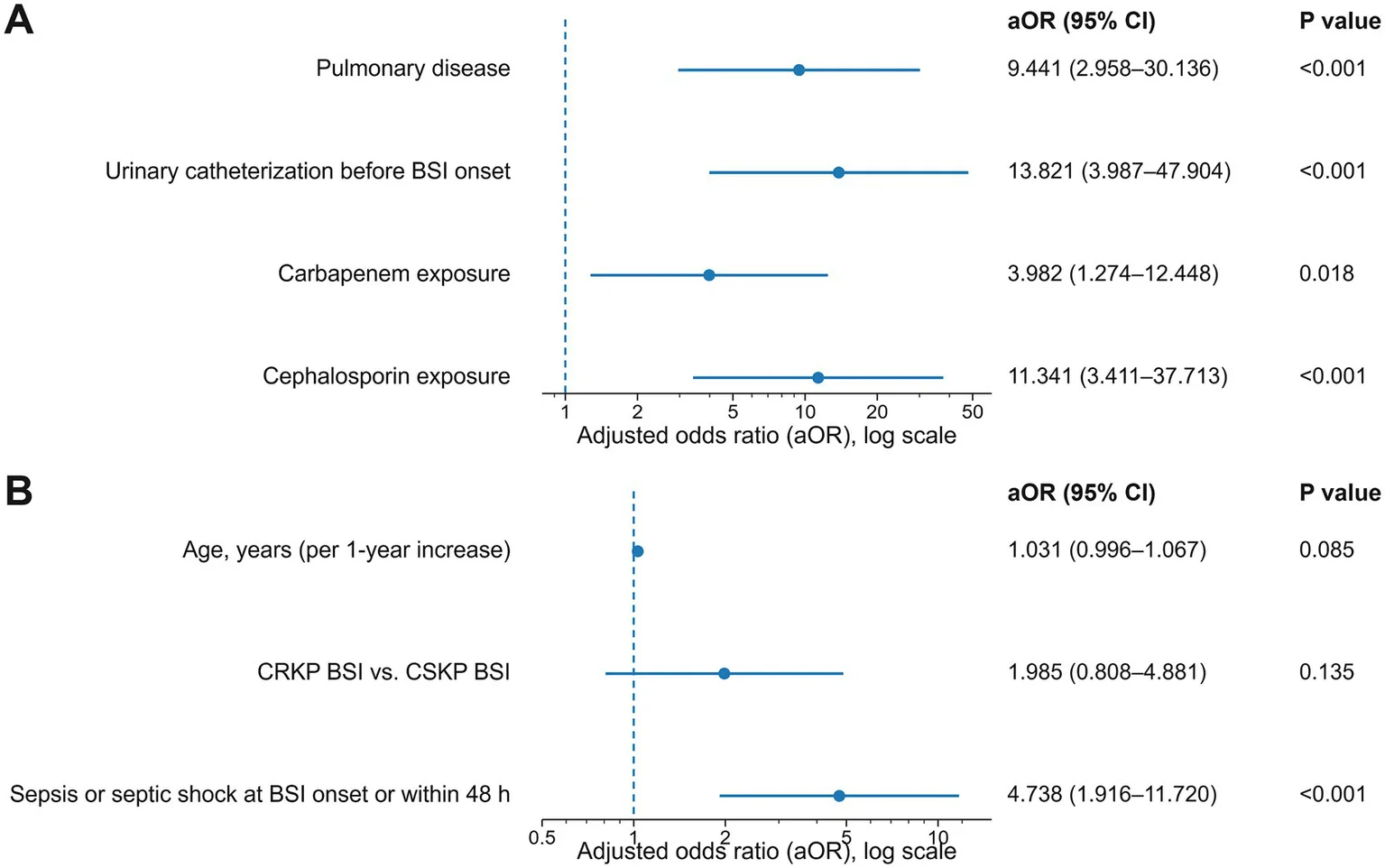
Forest plots of multivariable logistic regression analyses of **(A)** Factors associated with CRKP BSI compared with CSKP BSI and **(B)** factors associated with in-hospital all-cause mortality among patients with *K. pneumoniae* BSI. CRKP, carbapenem-resistant *Klebsiella pneumoniae*; CSKP, carbapenem-susceptible *Klebsiella pneumoniae*; BSI, bloodstream infection; aOR, adjusted odds ratio; CI, confidence interval.

In the Firth sensitivity analysis, the aORs were attenuated relative to the primary analysis, but all four associations remained positive, and their 95% confidence intervals excluded 1. The intervals remained wide, indicating limited precision of the effect estimates (Supplementary Table S4).

#### Factors associated with in-hospital all-cause mortality in *Klebsiella pneumoniae* BSI

Univariable comparisons in the overall *K. pneumoniae* BSI cohort identified several differences between survivors and non-survivors (Table 2). Non-survivors were older (median, 73 vs. 69 years; *p* = 0.006) and more likely to have clinician-documented sepsis or septic shock at BSI onset or within 48 h thereafter (54.3% vs. 17.0%; *p* < 0.001). CRKP BSI was also more common among non-survivors than among survivors (57.1% vs. 27.4%; *p* = 0.002). Several procedures initiated after BSI onset, including urinary catheterization, nasogastric tube placement, arterial or venous catheterization, and blood purification, were more common among non-survivors (all *p* < 0.05). These post-BSI procedures and the ICU length of stay during the index hospitalization were considered clinical-course variables and were not interpreted as baseline prognostic or causal factors.

In exploratory comparisons within the CRKP and CSKP subgroups, sepsis or septic shock was more common among non-survivors than among survivors (*p* = 0.010 and *p* = 0.048, respectively; Table 2). Given the small numbers of deaths in the CRKP and CSKP subgroups (20 and 15, respectively) and the large number of univariable comparisons, all subgroup findings were considered exploratory and hypothesis-generating; no separate multivariable mortality models were fitted for either subgroup.

In the overall multivariable mortality model, sepsis or septic shock was associated with higher odds of in-hospital all-cause mortality after adjustment for age and carbapenem-resistance phenotype (aOR, 4.738; 95% CI, 1.916–11.720; *p* < 0.001). The adjusted associations of CRKP BSI status (aOR, 1.985; 95% CI, 0.808–4.881; *p* = 0.135) and age (aOR per 1-year increase, 1.031; 95% CI, 0.996–1.067; *p* = 0.085) with mortality did not reach statistical significance (Supplementary Table S2; Figure 3B). The Hosmer–Lemeshow test did not detect a statistically significant lack of fit (*p* = 0.076). Across the final carbapenem-resistance and mortality models, no substantial multicollinearity was observed among the retained covariates (VIF range, 1.023–1.173).

## Discussion

National surveillance data from China have identified *K. pneumoniae* as a leading cause of BSI and documented a marked increase in the proportion of CRKP among *K. pneumoniae* bloodstream isolates (Chen Y. et al., 2022). Moreover, CRKP BSI is associated with significantly higher mortality than CSKP BSI (Li et al., 2023). Carbapenem resistance in *K. pneumoniae* is commonly mediated by carbapenemase production, including KPC, NDM, and OXA-48-like enzymes (Pitout et al., 2015). Non-carbapenemase-mediated resistance may also arise from the combined effects of ESBLs or AmpC β-lactamases and reduced outer-membrane permeability caused by porin alterations or loss (Pitout et al., 2015; Lee et al., 2024). These mechanisms provide important background for understanding the therapeutic challenge posed by CRKP infection; however, they were not directly investigated in this study. In this single-center retrospective cohort, we identified clinical factors associated with CRKP BSI compared with CSKP BSI, factors associated with in-hospital mortality, and described the local phenotypic antimicrobial susceptibility profile of CRKP bloodstream isolates.

Against this broader background, our single-center data showed lower annual proportions of *K. pneumoniae* BSI, hospital-acquired *K. pneumoniae* BSI, and CRKP BSI in the later study years. Given the small annual case counts, interpret these findings descriptively. The imipenem resistance rate among *K. pneumoniae* BSI isolates remained relatively high from 2019 to 2024, ranging from 20.0 to 45.5%, but decreased to 13.6% in 2025. These temporal patterns may reflect changes in patient mix, microbiological sampling, antimicrobial use, infection-prevention practices, and clinical workflows, including those during our hospital’s period as a designated Coronavirus disease 2019 (COVID-19) treatment center.

In this cohort of patients with *K. pneumoniae* BSI, prior cephalosporin and carbapenem exposure remained associated with CRKP rather than CSKP BSI after multivariable adjustment. Previous studies reported adjusted associations with carbapenem and tigecycline exposure (Li Y. et al., 2020) and with carbapenem and conventional BLBLI exposure (Cao et al., 2022). The carbapenem finding is also consistent with the multicenter study by Lou et al. (2022). These associations may partly reflect antibiotic selection pressure (Li Y. et al., 2020; Cao et al., 2022); however, residual confounding by treatment indication and prior healthcare exposures cannot be excluded. Because the comparison group comprised patients with CSKP BSI, interpret these associations within the population of patients with *K. pneumoniae* BSI. Tigecycline exposure was associated with CRKP BSI in univariable analysis, but only four patients were exposed, all in the CRKP group; therefore, it was excluded from the multivariable model, precluding assessment of an adjusted association. Collectively, these findings support careful review of broad-spectrum β-lactam prescribing, while the cephalosporin association warrants validation with more detailed exposure data.

Local susceptibility findings provide complementary information to support antimicrobial stewardship and treatment decisions. Among CRKP isolates, resistance to amikacin, doxycycline, and trimethoprim–sulfamethoxazole ranged from 61.2 to 71.4%, whereas susceptibility to ceftazidime/avibactam and tigecycline was 96.4% (27/28) and 95.9% (47/49), respectively. Results for ceftazidime/avibactam, colistin, minocycline, and doxycycline were available only for 2023–2025 and may not represent the full 2019–2025 CRKP cohort. All 28 colistin-tested isolates had VITEK 2 Compact MICs ≤2 mg/L, but confirmatory broth microdilution was not routinely performed, limiting interpretation. Zeng et al. reported resistance rates of 49.0% to amikacin and 64.7% to polymyxin B in an ICU outbreak investigation (Zeng et al., 2021). Differences in study populations, specimen sources, susceptibility-testing methods, and the polymyxin tested limit direct comparison with our findings and underscore the importance of locally generated susceptibility data.

The high *in vitro* susceptibility to ceftazidime/avibactam and tigecycline should be interpreted alongside clinical evidence and pharmacokinetic considerations. A nationwide survey of Chinese tertiary hospitals reported limited routine availability of ceftazidime/avibactam and frequent use of tigecycline-containing regimens (Zha et al., 2022). In a multicenter retrospective study from an OXA-48-endemic region, initiating ceftazidime/avibactam on the day of blood culture collection was associated with lower 30-day mortality among patients with ceftazidime/avibactam-susceptible CRKP BSI (Mert et al., 2024). For tigecycline, its clinical indications and relatively low serum concentrations warrant particular consideration in BSI (Stein and Babinchak, 2013; Rodvold et al., 2006; Falagas et al., 2009). A meta-analysis reported higher mortality with polymyxin monotherapy than with polymyxin-based combination therapy in CRKP BSI (Hou et al., 2020), while a retrospective cohort of patients with CRKP infections identified an association between baseline renal insufficiency and treatment failure during polymyxin B monotherapy (Dubrovskaya et al., 2013). These findings should be interpreted within their respective populations and treatment contexts and do not justify routine polymyxin-based combination therapy. Studies of tigecycline combinations using simulated pulmonary exposures (Wiskirchen et al., 2011), together with *in vitro* investigations of cefiderocol and plazomicin (Falagas et al., 2017; Galani et al., 2019), provide evidence of antibacterial activity but do not establish clinical effectiveness in BSI. Our study likewise did not evaluate the comparative effectiveness of treatment regimens. Local susceptibility findings should therefore be integrated with clinical evidence, infection-site considerations, and patient-specific factors to inform antimicrobial selection.

Beyond optimizing treatment, preventing CRKP BSI requires identifying clinical settings where modifiable risk factors converge. In our cohort, any ICU admission during the index hospitalization and BSI onset in the ICU were more common among patients with CRKP BSI than among those with CSKP BSI in univariable analyses, consistent with previous reports highlighting the ICU as an important setting for CRKP infection, including BSI caused by pandrug-resistant strains (Cheng et al., 2024; Alicino et al., 2015; Chen J. et al., 2022; Papadimitriou-Olivgeris et al., 2021; Li J et al., 2020). The former variable did not distinguish between pre-BSI and post-BSI ICU admissions, whereas the latter described the care setting at the time of BSI onset. These associations should be interpreted as markers of a high-risk care environment rather than as evidence of an independent causal effect of ICU care. The observed association may reflect the combined influence of greater illness severity, frequent use of invasive devices, higher antimicrobial selection pressure, and increased opportunities for healthcare-associated acquisition. In the ICU setting, multimodal infection-prevention measures may be particularly important, including active surveillance, device-associated infection-prevention bundles, rigorous hand hygiene and environmental cleaning, contact precautions, and antimicrobial stewardship (Cornistein et al., 2023).

Pulmonary disease remained associated with CRKP rather than CSKP BSI after multivariable adjustment. This association may reflect both host susceptibility and healthcare-related exposures. Impaired mucociliary clearance and altered host defenses in chronic pulmonary conditions could plausibly favor respiratory colonization or infection, and repeated antimicrobial exposure and airway instrumentation may also contribute. However, these mechanisms were not directly assessed, and the culture data could neither distinguish colonization from infection nor establish a respiratory source of BSI. These potential explanations provide context for examining associations between invasive-device use before BSI onset and CRKP BSI.

Previous studies have linked catheter use to CRKP infection, including intravascular catheterization in the study by Lou et al. (2022) and indwelling catheter use in a meta-analysis (Li J. et al., 2020). In our cohort, urinary catheterization, nasogastric tube placement, and arterial or venous catheterization before BSI onset were associated with CRKP rather than CSKP BSI in univariable analysis. The association with urinary catheterization persisted after multivariable adjustment. This association may partly reflect differences in illness severity, catheterization indications, or accompanying healthcare exposures. Without clinically adjudicated infection-source data, we could not determine whether BSI originated from the urinary tract, and the observed association does not establish causality. These findings nevertheless underscore the importance of appropriate urinary catheter use, including careful assessment of indications, standardized insertion and maintenance, and prompt removal when no longer clinically necessary.

In our univariable analysis, open surgery performed within 3 months before BSI onset was associated with CRKP rather than CSKP BSI, whereas non-open surgical procedures were not. Although previous studies have identified surgery as a risk factor for CRKP infection, they did not distinguish among different surgical approaches (Li Y. et al., 2020; Li J. et al., 2020; Kofteridis et al., 2014). A meta-analysis of randomized trials in colorectal surgery found that laparoscopic procedures were associated with a lower incidence of surgical site infection than open procedures (Kulkarni and Arulampalam, 2020). However, the meta-analysis was limited to surgical site infections after colorectal surgery and cannot be directly extrapolated to CRKP BSI or used to establish a causal relationship between open surgery and CRKP BSI. Moreover, the multivariable model for CRKP BSI did not retain open surgery, and residual confounding by operative complexity, postoperative healthcare exposure, invasive-device use, and antimicrobial exposure cannot be excluded. Therefore, this association should be regarded as exploratory. Rather than indicating an independent effect of open surgery, this finding supports continued attention to established perioperative infection-prevention practices, including appropriate wound and device management and prudent antimicrobial stewardship, among surgical patients at risk for CRKP infection.

Li et al. found that enema administration was associated with CRKP BSI in univariable analysis but did not identify it as an independent risk factor (Li Y. et al., 2020). Consistent with their findings, our univariable analysis showed an association between pre-BSI enema exposure and CRKP rather than CSKP BSI. This exploratory association may reflect differences in enema indications, underlying illness, and concurrent healthcare exposures. The potential roles of altered intestinal mucosal integrity and changes in intestinal colonization remain speculative, as neither was directly assessed in our study. Further multicenter studies incorporating detailed clinical exposure data and intestinal colonization surveillance are needed to validate this association and explore possible underlying mechanisms.

Non-blood-site *K. pneumoniae* detection was more frequent among patients with CRKP than among those with CSKP BSI, including detection before BSI onset and at multiple sites. This pattern may reflect colonization, localized infection, or differences in clinical sampling. Among patients who underwent sputum culture, positivity was higher in the CRKP group; however, clinician-directed sampling limits inference about respiratory tropism. Previous research has associated non-blood-site CRKP detection with CRKP BSI (Cheng et al., 2024). The gastrointestinal tract is a well-known reservoir for *K. pneumoniae* colonization (Martin and Bachman, 2018), and respiratory tract colonization has also been reported (Fang et al., 2025). Colonization may precede invasive infection (Zhang et al., 2025; Bray and Zafar, 2024). However, our study could not distinguish colonization from localized infection or establish clonal relatedness between non-blood-site and bloodstream isolates. These findings therefore do not identify a specific source of BSI or demonstrate spread from a non-blood site to the bloodstream. Positive non-blood-site cultures may nevertheless inform clinical assessment, including evaluation of potential infection sources, review of invasive-device use, and consideration of appropriate infection-prevention measures.

In-hospital mortality among patients with *K. pneumoniae* BSI was 24.8% in our cohort, a figure numerically similar to the crude 30-day mortality of 26.39% reported in a Shanghai study (Xiao et al., 2024). Previous research has documented substantial mortality among patients with CRKP infection (Xu et al., 2017), and a meta-analysis reported higher mortality with carbapenem-resistant compared with carbapenem-susceptible Enterobacteriaceae infections (Falagas et al., 2014). In our cohort, crude in-hospital mortality was higher among patients with CRKP than CSKP BSI (40.8% vs. 16.3%), consistent with Liang et al. (2022). However, Zheng et al. (2017) found no significant difference in 28-day mortality between the two groups in an ICU cohort. Differences in study populations and mortality endpoints limit direct comparisons across studies. Moreover, the crude mortality difference observed in our cohort does not establish an independent effect of carbapenem resistance.

Previous studies of nosocomial BSI and CRKP or carbapenemase-producing *K. pneumoniae* BSI have reported varying associations between mortality and patient characteristics, illness severity, pathogen characteristics, and treatment-related factors (Chen J. et al., 2022; Zheng et al., 2017; Reunes et al., 2011; Nutman et al., 2022; Papadimitriou-Olivgeris et al., 2017). In the present cohort, sepsis or septic shock assessed at BSI onset or within 48 h thereafter was associated with higher odds of in-hospital all-cause mortality after adjustment for age and CRKP BSI status. The adjusted association between CRKP BSI status and mortality did not reach statistical significance (aOR, 1.985; 95% CI, 0.808–4.881; *p* = 0.135), and the wide confidence interval indicates limited precision. The present mortality analysis did not account for concordance between empirical antimicrobial therapy and subsequent susceptibility results or the timing of effective antimicrobial therapy, limiting interpretation of the adjusted association between carbapenem resistance and in-hospital mortality. With only 35 deaths overall, the mortality model was restricted to three clinically selected covariates: age, CRKP BSI status, and sepsis or septic shock. Given the small number of deaths in the CRKP and CSKP groups (20 and 15, respectively), no subgroup-specific multivariable models were fitted. Subgroup findings should be considered exploratory because of the small event counts and the multiple univariable comparisons.

In univariable analysis, initiating arterial or venous catheterization after BSI onset was associated with in-hospital mortality. However, because post-BSI catheterization may be performed in response to clinical deterioration, hemodynamic instability, or escalating supportive-care needs, this association may reflect confounding by indication and time-varying illness severity rather than a direct effect of catheterization. It should therefore be interpreted as a clinical-course association and a potential marker of illness severity and intensity of care, rather than as a causal or baseline prognostic factor. Taken together, these findings underscore the multifactorial nature of in-hospital mortality among patients with *K. pneumoniae* BSI and highlight the need for validation in larger multicenter cohorts.

## Conclusion

CRKP BSI remains a substantial clinical and infection-control challenge, given its higher crude in-hospital mortality than CSKP BSI and limited therapeutic options. In this single-center cohort of patients with *K. pneumoniae* BSI, pulmonary disease, urinary catheterization before BSI onset, prior carbapenem exposure, and prior cephalosporin exposure were associated with carbapenem resistance in multivariable analysis. In the mortality analysis, sepsis or septic shock assessed at BSI onset or within 48 h thereafter was associated with higher odds of in-hospital all-cause mortality after adjustment for age and CRKP BSI status. However, the adjusted association between CRKP BSI status and mortality did not reach statistical significance. These findings support antimicrobial stewardship, appropriate urinary catheter use, targeted infection-prevention measures, and early recognition and management of sepsis.

## Limitations

This study has several limitations. First, as a single-center retrospective study, our findings may be less generalizable to other healthcare settings, particularly those with different patient demographics, antimicrobial stewardship strategies, infection control policies, or local epidemiology of CRKP.

Second, the limited cohort size constrained model complexity and estimation precision, and only 35 deaths further limited the mortality analysis. Annual estimates should be interpreted descriptively, and subgroup comparisons should be treated as exploratory. Although the Firth sensitivity analysis yielded associations consistent in direction with the original CRKP model, the confidence intervals remained wide, indicating uncertainty about their magnitude. The reported confidence intervals do not account for uncertainty arising from univariable screening and clinical covariate selection. Because the Firth analysis retained the same covariates, it did not evaluate this selection uncertainty.

Third, incomplete clinical data and limitations in diagnostic ascertainment constrained adjustment for relevant clinical factors. APACHE II scores were available for only 43 of 141 patients and were therefore excluded from the primary mortality model. Sepsis or septic shock was identified from clinician-documented diagnoses without systematic retrospective adjudication using Sepsis-3 criteria. Variation in diagnostic and documentation practices across departments and over the seven-year study period may have introduced misclassification, affecting both the estimated association of sepsis or septic shock with mortality and adjustment for early illness severity. The direction and magnitude of any resulting bias are uncertain. The analytical dataset lacked a clinically adjudicated primary source of BSI, leaving potential residual confounding by infection source in both models; non-blood-site culture positivity could not substitute for this information. Although treatment regimens were collected, patient-level concordance between empirical therapy and subsequent susceptibility results was not evaluated. Neither this concordance nor the timing of effective therapy was included in the mortality model, limiting interpretation of the association between carbapenem resistance and in-hospital mortality. ICU length of stay included post-BSI time and could be influenced by subsequent illness severity and the timing of ICU discharge or death. These descriptive comparisons were limited to ICU-admitted patients and should not be interpreted as evidence of a pre-BSI risk factor, baseline prognostic value, or a causal effect.

Fourth, carbapenemase characterization was not systematically performed, and molecular typing and whole-genome sequencing were unavailable, limiting the assessment of resistance mechanisms and the determination of clonal relatedness between bloodstream and non-blood-site isolates. Non-blood-site sampling was clinician-directed rather than standardized, potentially introducing selection bias and limiting the interpretation of site-specific positivity rates. The culture findings could not reliably distinguish colonization from localized infection or establish a direct microbiological link between non-blood-site detection and BSI.

Finally, ceftazidime/avibactam, colistin, minocycline, and doxycycline were tested only during 2023–2025, with results available for 28 of 49 CRKP isolates per agent. This limited testing period may introduce potential selection bias and limits generalizability to the full 2019–2025 CRKP cohort, particularly earlier isolates. Colistin MICs were determined using the VITEK 2 Compact system without routine confirmation by broth microdilution; therefore, interpret these MIC results cautiously. Larger prospective multicenter studies incorporating standardized severity assessment, infection-source adjudication, treatment documentation, and microbiological characterization are warranted.

## Data Availability

The original contributions presented in the study are included in the article/Supplementary material, further inquiries can be directed to the corresponding author.

## References

[ref1] Alicino C. Giacobbe D. R. Orsi A. Tassinari F. Trucchi C. Sarteschi G. . (2015). Trends in the annual incidence of carbapenem-resistant *Klebsiella pneumoniae* bloodstream infections: a 8-year retrospective study in a large teaching hospital in northern Italy. BMC Infect. Dis. 15:415. doi: 10.1186/s12879-015-1152-0, 26464061 PMC4605101

[ref2] Bray A. S. Zafar M. A. (2024). Deciphering the gastrointestinal carriage of *Klebsiella pneumoniae*. Infect. Immun. 92:e0048223. doi: 10.1128/iai.00482-23, 38597634 PMC11384780

[ref3] Cao Z. Yue C. Kong Q. Liu Y. Li J. (2022). Risk factors for a hospital-acquired Carbapenem-resistant *Klebsiella pneumoniae* bloodstream infection: a five-year retrospective study. Infect. Drug Resist. 15, 641–654. doi: 10.2147/IDR.S342103, 35241916 PMC8887613

[ref4] Chen Y. Ji J. Ying C. Liu Z. Yang Q. Kong H. . (2022). Blood bacterial resistant investigation collaborative system (BRICS) report: a national surveillance in China from 2014 to 2019. Antimicrob. Resist. Infect. Control 11:17. doi: 10.1186/s13756-022-01055-5 PMC8785473 35074014

[ref5] Chen J. Ma H. Huang X. Cui Y. Peng W. Zhu F. . (2022). Risk factors and mortality of carbapenem-resistant *Klebsiella pneumoniae* bloodstream infection in a tertiary-care hospital in China: an eight-year retrospective study. Antimicrob. Resist. Infect. Control 11:161. doi: 10.1186/s13756-022-01204-w, 36536423 PMC9761986

[ref6] Cheng Y. Cheng Q. Zhang R. Gao J.-y. Li W. Wang F.-k. . (2024). Retrospective analysis of molecular characteristics, risk factors, and outcomes in carbapenem-resistant *Klebsiella pneumoniae* bloodstream infections. BMC Microbiol. 24:309. doi: 10.1186/s12866-024-03465-4, 39174950 PMC11340057

[ref7] Cornistein W. Santonato D. Novau P. A. Fabbro L. G. Jorge M. F. Malvicini M. A. . (2023). Synergy between infection control and antimicrobial stewardship programs to control carbapenem-resistant Enterobacterales. Antimicrob. Steward Healthc. Epidemiol. 3:e162. doi: 10.1017/ash.2023.439, 37771737 PMC10523542

[ref8] Dubrovskaya Y. Chen T. Y. Scipione M. R. Esaian D. Phillips M. S. Papadopoulos J. . (2013). Risk factors for treatment failure of polymyxin B monotherapy for carbapenem-resistant *Klebsiella pneumoniae* infections. Antimicrob. Agents Chemother. 57, 5394–5397. doi: 10.1128/AAC.00510-13, 23959321 PMC3811259

[ref9] Falagas M. E. Karageorgopoulos D. E. Dimopoulos G. (2009). Clinical significance of the pharmacokinetic and pharmacodynamic characteristics of tigecycline. Curr. Drug Metab. 10, 13–21. doi: 10.2174/138920009787048356, 19149509

[ref10] Falagas M. E. Skalidis T. Vardakas K. Z. Legakis N. J. Hellenic Cefiderocol Study Group (2017). Activity of cefiderocol (S-649266) against carbapenem-resistant gram-negative bacteria collected from inpatients in Greek hospitals. J. Antimicrob. Chemother. 72, 1704–1708. doi: 10.1093/jac/dkx049 28369471

[ref11] Falagas M. E. Tansarli G. S. Karageorgopoulos D. E. Vardakas K. Z. (2014). Deaths attributable to carbapenem-resistant Enterobacteriaceae infections. Emerg. Infect. Dis. 20, 1170–1175. doi: 10.3201/eid2007.121004, 24959688 PMC4073868

[ref12] Falcone M. Tiseo G. Carbonara S. Marino A. Di Caprio G. Carretta A. . (2023). Mortality attributable to bloodstream infections caused by different carbapenem-resistant gram-negative bacilli: results from a nationwide study in Italy (ALARICO network). Clin. Infect. Dis. 76, 2059–2069. doi: 10.1093/cid/ciad100, 36801828

[ref13] Fang H. Chen Y. Chen Y. Qi Y. Yan R. Guo F. (2025). In-host co-colonization and bloodstream infection by distinct classical and hypervirulent CRKP clones harboring a homologous *bla*_KPC-2_-harboring plasmid. Front. Cell. Infect. Microbiol. 15:1683743. doi: 10.3389/fcimb.2025.1683743, 41488477 PMC12757419

[ref14] Galani I. Nafplioti K. Adamou P. Karaiskos I. Giamarellou H. Souli M. (2019). Nationwide epidemiology of carbapenem resistant *Klebsiella pneumoniae* isolates from Greek hospitals, with regards to plazomicin and aminoglycoside resistance. BMC Infect. Dis. 19:167. doi: 10.1186/s12879-019-3801-1, 30770727 PMC6377745

[ref15] Hafiz T. A. Alanazi S. Alghamdi S. S. . (2023). *Klebsiella pneumoniae* bacteraemia epidemiology: resistance profiles and clinical outcome of king Fahad Medical City isolates, Riyadh, Saudi Arabia. BMC Infect. Dis. 23:579. doi: 10.1186/s12879-023-08563-8, 37670240 PMC10481561

[ref16] Hou S. Y. Wu D. Feng X. H. (2020). Polymyxin monotherapy versus polymyxin-based combination therapy against carbapenem-resistant *Klebsiella pneumoniae*: a systematic review and meta-analysis. J. Glob. Antimicrob. Resist. 23, 197–202. doi: 10.1016/j.jgar.2020.08.024 33091620

[ref17] Hovan M. R. Narayanan N. Cedarbaum V. Bhowmick T. Kirn T. J. (2021). Comparing mortality in patients with carbapenemase-producing carbapenem resistant Enterobacterales and non-carbapenemase-producing carbapenem resistant Enterobacterales bacteremia. Diagn. Microbiol. Infect. Dis. 101:115505. doi: 10.1016/j.diagmicrobio.2021.115505, 34399381

[ref18] Kofteridis D. P. Valachis A. Dimopoulou D. Maraki S. Christidou A. Mantadakis E. . (2014). Risk factors for carbapenem-resistant *Klebsiella pneumoniae* infection/colonization: a case-case-control study. J. Infect. Chemother. 20, 293–297. doi: 10.1016/j.jiac.2013.11.007, 24703709

[ref19] Kulkarni N. Arulampalam T. (2020). Laparoscopic surgery reduces the incidence of surgical site infections compared to the open approach for colorectal procedures: a meta-analysis. Tech. Coloproctol. 24, 1017–1024. doi: 10.1007/s10151-020-02293-8, 32648141 PMC7346580

[ref20] Lee Y. Q. Sri La Sri Ponnampalavanar S. Wong J. H. Kong Z. X. Ngoi S. T. Karunakaran R. . (2024). Investigation on the mechanisms of carbapenem resistance among the non-carbapenemase-producing carbapenem-resistant *Klebsiella pneumoniae*. Front. Cell. Infect. Microbiol. 14:1464816. doi: 10.3389/fcimb.2024.1464816 PMC11445613 39359938

[ref21] Li D. Huang X. Rao H. Yu H. Long S. Li Y. . (2023). *Klebsiella pneumoniae* bacteremia mortality: a systematic review and meta-analysis. Front. Cell. Infect. Microbiol. 13:1157010. doi: 10.3389/fcimb.2023.1157010, 37153146 PMC10159367

[ref22] Li Y. Li J. Hu T. Hu J. Song N. Zhang Y. . (2020). Five-year change of prevalence and risk factors for infection and mortality of carbapenem-resistant *Klebsiella pneumoniae* bloodstream infection in a tertiary hospital in North China. Antimicrob. Resist. Infect. Control 9:79. doi: 10.1186/s13756-020-00728-3, 32487221 PMC7268443

[ref23] Li J. Li Y. Song N. Chen Y. (2020). Risk factors for carbapenem-resistant *Klebsiella pneumoniae* infection: a meta-analysis. J. Glob. Antimicrob. Resist. 21, 306–313. doi: 10.1016/j.jgar.2019.09.006, 31525540

[ref24] Liang X. Chen P. Deng B. Sun F. H. Yang Y. Yang Y. . (2022). Outcomes and risk factors of bloodstream infections caused by Carbapenem-resistant and non-Carbapenem-resistant *Klebsiella pneumoniae* in China. Infect. Drug Resist. 15, 3161–3171. doi: 10.2147/IDR.S367588, 35747334 PMC9212785

[ref25] Lou T. Du X. Zhang P. Shi Q. Han X. Lan P. . (2022). Risk factors for infection and mortality caused by carbapenem-resistant *Klebsiella pneumoniae*: a large multicentre case-control and cohort study. J. Infect. 84, 637–647. doi: 10.1016/j.jinf.2022.03.010, 35301013

[ref26] Martin R. M. Bachman M. A. (2018). Colonization, infection, and the accessory genome of *Klebsiella pneumoniae*. Front. Cell. Infect. Microbiol. 8:4. doi: 10.3389/fcimb.2018.00004, 29404282 PMC5786545

[ref27] Mert A. Derin O. Akalın H. Dumlu R. Gündeş S. Zengin R. . (2024). Multicenter evaluation of ceftazidime-avibactam use in carbapenem-resistant *Klebsiella pneumoniae* bloodstream infections in OXA-48 endemic regions. Sci. Rep. 14:26337. doi: 10.1038/s41598-024-77259-z, 39487308 PMC11530633

[ref28] Nutman A. Wullfhart L. Temkin E. . (2022). Hospital-onset bloodstream infections caused by eight sentinel Bacteria: a Nationwide study in Israel, 2018-2019. Microorganisms 10:1009. doi: 10.3390/microorganisms10051009, 35630452 PMC9147328

[ref29] Papadimitriou-Olivgeris M. Bartzavali C. Georgakopoulou A. . (2021). Mortality of Pandrug-resistant *Klebsiella pneumoniae* bloodstream infections in critically ill patients: a retrospective cohort of 115 episodes. Antibiotics (Basel) 10:76. doi: 10.3390/antibiotics10010076, 33467394 PMC7830393

[ref30] Papadimitriou-Olivgeris M. Fligou F. Bartzavali C. . (2017). Carbapenemase-producing *Klebsiella pneumoniae* bloodstream infection in critically ill patients: risk factors and predictors of mortality. Eur. J. Clin. Microbiol. Infect. Dis. 36, 1125–1131. doi: 10.1007/s10096-017-2899-6 28102514

[ref31] Pitout J. D. Nordmann P. Poirel L. (2015). Carbapenemase-producing *Klebsiella pneumoniae*, a key pathogen set for global nosocomial dominance. Antimicrob. Agents Chemother. 59, 5873–5884. doi: 10.1128/AAC.01019-15 PMC4576115 26169401

[ref32] Reunes S. Rombaut V. Vogelaers D. Brusselaers N. Lizy C. Cankurtaran M. . (2011). Risk factors and mortality for nosocomial bloodstream infections in elderly patients. Eur. J. Intern. Med. 22, e39–e44. doi: 10.1016/j.ejim.2011.02.004, 21925041

[ref33] Rodvold K. A. Gotfried M. H. Cwik M. Korth-Bradley J. M. Dukart G. Ellis-Grosse E. J. (2006). Serum, tissue and body fluid concentrations of tigecycline after a single 100 mg dose. J. Antimicrob. Chemother. 58, 1221–1229. doi: 10.1093/jac/dkl403 17012300

[ref34] Stein G. E. Babinchak T. (2013). Tigecycline: an update. Diagn. Microbiol. Infect. Dis. 75, 331–336. doi: 10.1016/j.diagmicrobio.2012.12.004, 23357291

[ref35] Wiskirchen D. E. Koomanachai P. Nicasio A. M. Nicolau D. P. Kuti J. L. (2011). In vitro pharmacodynamics of simulated pulmonary exposures of tigecycline alone and in combination against *Klebsiella pneumoniae* isolates producing a KPC carbapenemase. Antimicrob. Agents Chemother. 55, 1420–1427. doi: 10.1128/AAC.01253-10, 21282442 PMC3067181

[ref36] Xiao S. Zhou S. Cao H. Han L. Zhao S. Wang X. (2024). Incidence, antimicrobial resistance and mortality of *Klebsiella pneumoniae* bacteraemia in Shanghai, China, 2018-2022. Infect. Dis. 56, 1021–1030. doi: 10.1080/23744235.2024.2374980, 38963702

[ref37] Xu L. Sun X. Ma X. (2017). Systematic review and meta-analysis of mortality of patients infected with carbapenem-resistant *Klebsiella pneumoniae*. Ann. Clin. Microbiol. Antimicrob. 16:18. doi: 10.1186/s12941-017-0191-3, 28356109 PMC5371217

[ref38] Zeng L. Yang C. Zhang J. Hu K. Zou J. Li J. . (2021). An outbreak of Carbapenem-resistant *Klebsiella pneumoniae* in an intensive care unit of a major teaching Hospital in Chongqing, China. Front. Cell. Infect. Microbiol. 11:656070. doi: 10.3389/fcimb.2021.656070, 34150672 PMC8208809

[ref39] Zha L. Li S. Ren Z. Li X. Zhang D. Zou Y. . (2022). Clinical management of infections caused by carbapenem-resistant *Klebsiella pneumoniae* in critically ill patients: a nationwide survey of tertiary hospitals in mainland China. J. Infect. 84, e108–e110. doi: 10.1016/j.jinf.2022.03.023, 35367511

[ref40] Zhang X. Y. Zhang D. Zhang X. F. Zhang J. P. Tian S. F. Zhang X. (2025). Research progress on *Klebsiella pneumoniae* colonization and infection. Zhonghua Chuan Ran Bing Za Zhi. 43, 407–416. doi: 10.3760/cma.j.cn311365-20250704-00213

[ref41] Zheng X. Wang J. F. Xu W. L. Xu J. Hu J. (2017). Clinical and molecular characteristics, risk factors and outcomes of Carbapenem-resistant *Klebsiella pneumoniae* bloodstream infections in the intensive care unit. Antimicrob. Resist. Infect. Control 6:102. doi: 10.1186/s13756-017-0256-2, 29026535 PMC5625719

