# Supplementary material for: Clinical factors associated with carbapenem resistance and in-hospital mortality in Klebsiella pneumoniae bloodstream infection: a 7-year retrospective cohort study
Source: Front Microbiol. 2026 Oct 8;17:1872672. doi: 10.3389/fmicb.2026.1872672 (PMC13687263; doi:10.3389/fmicb.2026.1872672)
Supplement: Supplementary file 1 [file Table_1.DOCX]

**Table S1.** Multivariable Logistic Regression Analysis of Factors Associated with CRKP BSI Compared with CSKP BSI Among Patients with *K. pneumoniae* BSI

| **Variables** | ***P* value** | **aOR** | **95% CI** |
| --- | --- | --- | --- |
| Pulmonary disease | <0.001 | 9.441 | (2.958-30.136) |
| Urinary catheterization before BSI onset | <0.001 | 13.821 | (3.987-47.904) |
| Carbapenem exposure | 0.018 | 3.982 | (1.274-12.448) |
| Cephalosporin exposure | <0.001 | 11.341 | (3.411-37.713) |

**NOTE:** Model N = 141, including 49 patients with CRKP BSI and 92 with CSKP BSI (reference group). Exposure-by-outcome cell counts and results of the Firth penalized logistic regression sensitivity analysis are presented in Table S4.

**Abbreviations**: CRKP, carbapenem-resistant *Klebsiella pneumoniae*; CSKP, carbapenem-susceptible *Klebsiella pneumoniae*; BSI, bloodstream infection; aOR, adjusted odds ratio; CI, confidence interval.
