# Supplementary material for: Clinical factors associated with carbapenem resistance and in-hospital mortality in Klebsiella pneumoniae bloodstream infection: a 7-year retrospective cohort study
Source: Front Microbiol. 2026 Oct 8;17:1872672. doi: 10.3389/fmicb.2026.1872672 (PMC13687263; doi:10.3389/fmicb.2026.1872672)
Supplement: Supplementary file 2 [file Table_2.DOCX]

**Table S2.** Multivariable Logistic Regression Analysis of Factors Associated with In-Hospital All-Cause Mortality Among Patients with *K. pneumoniae* BSI

| **Variables** | ***P* value** | **aOR** | **95% CI** |
| --- | --- | --- | --- |
| CRKP BSI vs. CSKP BSI | 0.135 | 1.985 | (0.808-4.881) |
| Sepsis or septic shock | <0.001 | 4.738 | (1.916-11.720) |
| Age, years | 0.085 | 1.031 | (0.996-1.067) |

**Note:** The primary endpoint was in-hospital all-cause mortality. Model N = 141; mortality events = 35. The model included age (per 1-year increase), carbapenem-resistance phenotype (reference: CSKP BSI), and sepsis or septic shock assessed at BSI onset or within 48 h thereafter.

**Abbreviations**: CRKP, carbapenem-resistant *Klebsiella pneumoniae*; CSKP, carbapenem-susceptible *Klebsiella pneumoniae*; BSI, bloodstream infection; aOR, adjusted odds ratio; CI, confidence interval.
