# Supplementary material for: Clinical factors associated with carbapenem resistance and in-hospital mortality in Klebsiella pneumoniae bloodstream infection: a 7-year retrospective cohort study
Source: Front Microbiol. 2026 Oct 8;17:1872672. doi: 10.3389/fmicb.2026.1872672 (PMC13687263; doi:10.3389/fmicb.2026.1872672)
Supplement: Supplementary file 3 [file Table_3.DOCX]

**Table S3.** Patient-level non-blood-site *K. pneumoniae* detection among patients sampled at the corresponding site (exploratory)

| **Non-blood site** | **Overall** | **CRKP BSI** | **CSKP BSI** | ***P* value** |
| --- | --- | --- | --- | --- |
| Sputum | 27/45 (60.0%) | 22/28 (78.6%) | 5/17 (29.4%) | 0.002 |
| Bronchoalveolar lavage fluid | 5/7 (71.4%) | 2/2 (100.0%) | 3/5 (60.0%) | 1.000 |
| Urine | 25/61 (41.0%) | 14/31 (45.2%) | 11/30 (36.7%) | 0.605 |
| Other non-blood sites | 33/62 (53.2%) | 14/22 (63.6%) | 19/40 (47.5%) | 0.290 |

**Note:** Values are n/N (%), where n is the number of patients with *K. pneumoniae* detected at the corresponding site and N is the number with at least one culture collected from that site in each BSI group; each patient was counted once per site. Other non-blood sites comprised catheter, drainage fluid, pus, bile, secretion, pleural fluid, and ascitic fluid. Analyses were exploratory and were not adjusted for multiple comparisons. Because sampling was clinician-directed, the findings should not be interpreted as evidence of site tropism. Positive cultures could not distinguish colonization from infection or establish clonal relatedness.

**Abbreviations:** BSI, bloodstream infection; CRKP, carbapenem-resistant *Klebsiella pneumoniae*; CSKP, carbapenem-susceptible *Klebsiella pneumoniae*.
