# Supplementary material for: Clinical factors associated with carbapenem resistance and in-hospital mortality in Klebsiella pneumoniae bloodstream infection: a 7-year retrospective cohort study
Source: Front Microbiol. 2026 Oct 8;17:1872672. doi: 10.3389/fmicb.2026.1872672 (PMC13687263; doi:10.3389/fmicb.2026.1872672)
Supplement: Supplementary file 4 [file Table_4.DOCX]

**Table S4.** Exposure distributions and Firth penalized logistic regression sensitivity analysis for factors associated with CRKP BSI compared with CSKP BSI

| **Variables** | **CRKP BSI（n=49）Present / Absent, n** | **CSKP BSI（n=92）Present / Absent, n** | **Firth aOR（95% CI）** | ***P* value** |
| --- | --- | --- | --- | --- |
| Pulmonary disease | 31/18 | 21/71 | 8.063（2.860–26.585） | <0.001 |
| Urinary catheterization before BSI onset | 29/20 | 9/83 | 11.589（3.805–41.570） | <0.001 |
| Carbapenem exposure | 23/26 | 9/83 | 3.700（1.248–11.387） | 0.018 |
| Cephalosporin exposure | 28/21 | 12/80 | 9.652（3.275–33.089） | <0.001 |

**Note:** All four predictors were entered simultaneously in the Firth model. The 95% CIs and *P* values were based on penalized profile likelihood and penalized likelihood-ratio tests, respectively.

**Abbreviations:** CRKP, carbapenem-resistant *Klebsiella pneumoniae*; CSKP, carbapenem-susceptible *Klebsiella pneumoniae*; BSI, bloodstream infection; aOR, adjusted odds ratio; CI, confidence interval.
