# Supplementary material for: Clinical factors associated with carbapenem resistance and in-hospital mortality in Klebsiella pneumoniae bloodstream infection: a 7-year retrospective cohort study
Source: Front Microbiol. 2026 Oct 8;17:1872672. doi: 10.3389/fmicb.2026.1872672 (PMC13687263; doi:10.3389/fmicb.2026.1872672)
Supplement: Supplementary file 5 [file Table__5.docx]

Table S5. Covariate selection for the CRKP-versus-CSKP model and the overall in-hospital mortality model

| **Model** | **Variable assessed** | **Univariable *P* value** | **Included in final model** | **Reason for exclusion or retention** |
| --- | --- | --- | --- | --- |
| CRKP vs. CSKP | Age | 0.028 | No | Met univariable screening, but was not retained in the restricted model because specific, clinically interpretable exposure domains were prioritized given the limited sample size. Residual confounding by age cannot be excluded. |
| CRKP vs. CSKP | Total length of stay, days | <0.001 | No | Excluded from the CRKP-versus-CSKP model because total length of stay included post-BSI time and could be influenced by BSI and its subsequent clinical course; it did not represent a strictly pre-BSI exposure. |
| CRKP vs. CSKP | Time from admission to BSI onset, days | <0.001 | No | Pre-BSI hospitalization duration met the univariable screening criterion but was not retained following clinical prioritization of specific device and antibiotic exposures in the restricted CRKP model. Residual confounding related to the duration of hospitalization before BSI remains possible. |
| CRKP vs. CSKP | Pulmonary disease | <0.001 | Yes | Retained as a clinically relevant baseline comorbidity and an a priori clinical domain of interest; mechanistic pathways were not directly assessed. |
| CRKP vs. CSKP | Solid tumor | 0.010 | No | Met univariable screening but was not retained because other clinically specific covariates were prioritized to limit model complexity. Residual confounding by tumor status remains possible. |
| CRKP vs. CSKP | Sepsis or septic shock | <0.001 | No | Excluded from the CRKP-versus-CSKP model because it was assessed at BSI onset or within 48 h thereafter and represented early illness severity rather than a pre-BSI exposure. It was retained as an early severity indicator in the mortality model. |
| CRKP vs. CSKP | Hospitalization within 3 months | 0.004 | No | Met univariable screening but was not retained because specific pre-BSI device and antimicrobial exposures were prioritized. Residual confounding related to prior healthcare exposure remains possible. |
| CRKP vs. CSKP | Any ICU admission during the index hospitalization | <0.001 | No | Excluded because it combined ICU admissions before and after BSI onset without distinguishing their timing and therefore did not consistently represent a pre-BSI exposure. |
| CRKP vs. CSKP | BSI onset in the ICU | <0.001 | No | BSI onset in the ICU was considered a care-setting characteristic at the index time point but was not retained after clinical prioritization of the selected covariates to limit model complexity. Residual confounding related to ICU care and underlying illness severity remains possible. |
| CRKP vs. CSKP | ICU length of stay during the index hospitalization, days | 0.025 | No | Not included in the reported final model. The measurement encompassed post-BSI time and did not represent a strictly pre-BSI exposure; the comparison was retained only as a descriptive clinical-course analysis among ICU-admitted patients. |
| CRKP vs. CSKP | Urinary catheterization before BSI onset | <0.001 | Yes | Retained as a clinically relevant pre-BSI device exposure within the a priori domain of invasive procedures; urinary catheterization was prioritized during clinical covariate selection. |
| CRKP vs. CSKP | Nasogastric tube placement before BSI onset | <0.001 | No | Pre-BSI nasogastric tube placement was not retained because urinary catheterization was clinically prioritized to limit device-related covariates. These are distinct exposures; the decision reflected clinical judgment rather than demonstrated multicollinearity. |
| CRKP vs. CSKP | Arterial or venous catheterization before BSI onset | <0.001 | No | Pre-BSI vascular catheterization was not retained because urinary catheterization was clinically prioritized to limit device-related covariates. These are distinct exposures, and residual confounding related to vascular catheterization remains possible. |
| CRKP vs. CSKP | Blood purification before BSI onset | 0.002 | No | Pre-BSI blood purification was not retained because pulmonary disease and selected device and antimicrobial exposures were clinically prioritized to limit model complexity. Residual confounding related to blood purification remains possible. |
| CRKP vs. CSKP | Invasive respiratory support before BSI onset | <0.001 | No | Pre-BSI invasive respiratory support was not retained because pulmonary disease and selected device and antimicrobial exposures were clinically prioritized to limit model complexity. Residual confounding related to respiratory support remains possible. |
| CRKP vs. CSKP | Enema before BSI onset | 0.003 | No | Pre-BSI enema was not retained because only 15 patients were exposed (CSKP, 4; CRKP, 11), and selected device and antimicrobial exposures were clinically prioritized to limit model complexity. |
| CRKP vs. CSKP | Open surgery within 3 months before BSI onset | 0.003 | No | Open surgery within 3 months before BSI onset was not retained because only 15 patients were exposed (CSKP, 4; CRKP, 11), and pulmonary disease and selected device and antimicrobial exposures were clinically prioritized to limit model complexity. |
| CRKP vs. CSKP | Puncture before BSI onset | 0.05 | No | Pre-BSI puncture met the screening criterion based on the unrounded *P* value, but only seven patients were exposed (CSKP, 2; CRKP, 5). It was not retained because of sparse exposure data and the need to limit model complexity. |
| CRKP vs. CSKP | Invasive procedures ≥ 3 before BSI onset | <0.001 | No | This composite indicator included urinary catheterization among the procedures counted. Pre-BSI urinary catheterization was prioritized as a specific, clinically interpretable exposure to limit model complexity. |
| CRKP vs. CSKP | Any antibiotic exposure | <0.001 | No | The “any antibiotic exposure” indicator included the retained carbapenem and cephalosporin exposures. These class-specific variables were prioritized to assess their respective associations with CRKP BSI and limit model complexity. |
| CRKP vs. CSKP | Carbapenem exposure | <0.001 | Yes | Retained as a clinically relevant pre-BSI antimicrobial exposure to assess its association with carbapenem resistance among patients with *K. pneumoniae* BSI. |
| CRKP vs. CSKP | Conventional BLBLIs exposure | <0.001 | No | Conventional BLBLI exposure was not retained because only 17 patients were exposed (CSKP, 4; CRKP, 13), and carbapenem and cephalosporin exposures were clinically prioritized to limit antibiotic-class covariates. |
| CRKP vs. CSKP | Cephalosporin exposure | <0.001 | Yes | Retained as a clinically relevant pre-BSI antimicrobial exposure to assess its association with CRKP BSI while adjusting for carbapenem exposure and the other retained covariates. |
| CRKP vs. CSKP | Quinolones exposure | <0.001 | No | Only 17 patients had quinolone exposure (CSKP, 4; CRKP, 13). Given the small exposed subgroup and the need to limit antibiotic-class covariates, carbapenem and cephalosporin exposures were clinically prioritized. |
| CRKP vs. CSKP | Glycopeptides exposure | 0.033 | No | Only 10 patients had glycopeptide exposure (CSKP, 3; CRKP, 7). Given the small exposed subgroup and the need to limit antibiotic-class covariates, carbapenem and cephalosporin exposures were clinically prioritized. |
| CRKP vs. CSKP | Tigecycline exposure | 0.013 | No | Only four patients were exposed, all in the CRKP group (CSKP, 0; CRKP, 4). Sparse exposure and a zero cell could compromise conventional maximum-likelihood estimation; the variable was therefore not retained in the restricted model. |
| CRKP vs. CSKP | Non-blood-site *K. pneumoniae* detection | <0.001 | No | The overall non-blood-site detection variable in Table 4 combined detections before, on the day of, and after BSI onset (Table 5). It was excluded from the CRKP-versus-CSKP model because it was not restricted to pre-BSI detection. |
| Mortality | Age, years | 0.006 | Yes | Retained as a clinically relevant demographic covariate and modeled continuously to adjust for age differences. Retention was not conditional on statistical significance in the final model. |
| Mortality | Time from admission to BSI onset, days | 0.005 | No | Although temporally eligible, pre-BSI hospitalization duration was not retained because age, resistance phenotype, and early illness severity were clinically prioritized to limit model complexity given the 35 deaths. Residual confounding related to pre-BSI hospitalization duration remains possible. |
| Mortality | Sepsis or septic shock | <0.001 | Yes | Retained as a clinician-documented indicator of early illness severity, assessed at BSI onset or within 48 h thereafter. |
| Mortality | Any ICU admission during the index hospitalization | 0.006 | No | Excluded because it combined ICU admissions before and after BSI onset without distinguishing their timing and could reflect the subsequent clinical course rather than the care setting at BSI onset. |
| Mortality | BSI onset in the ICU | <0.001 | No | Given the limited number of deaths, age, resistance phenotype, and early illness severity were clinically prioritized to limit model complexity; BSI onset in the ICU was therefore not retained. Residual confounding related to ICU care remains possible. |
| Mortality | Arterial or venous catheterization before BSI onset | 0.005 | No | Eligible pre-BSI exposure, but not prioritized over age, resistance phenotype, and early illness severity in the restricted three-covariate model. Residual confounding remains possible. |
| Mortality | Urinary catheterization after BSI onset | 0.045 | No | Post-BSI clinical-course variable. Excluded to avoid interpreting a potentially time-dependent consequence or marker of illness severity as a baseline prognostic factor. |
| Mortality | Nasogastric tube placement after BSI onset | <0.001 | No | Post-BSI clinical-course variable; excluded because it may reflect evolving illness severity and intensity of care. |
| Mortality | Arterial or venous catheterization after BSI onset | <0.001 | No | Post-BSI clinical-course variable; excluded because it may be initiated in response to deterioration, hemodynamic instability, or escalating supportive care. |
| Mortality | Blood purification after BSI onset | 0.002 | No | Post-BSI clinical-course variable; excluded because initiation may reflect subsequent organ dysfunction or clinical deterioration. |
| Mortality | ≥ 3 types of invasive procedures after BSI onset | <0.001 | No | Post-BSI composite clinical-course measure; excluded because it may reflect evolving illness severity and intensity of care rather than a baseline exposure. |
| Mortality | Carbapenem exposure | <0.001 | No | Prior carbapenem exposure was eligible for consideration but was not prioritized over age, resistance phenotype, and early illness severity in the restricted mortality model. Residual confounding related to prior carbapenem exposure remains possible. |
| Mortality | Quinolones exposure | 0.035 | No | Prior quinolone exposure was eligible for consideration but was not prioritized over age, resistance phenotype, and early illness severity in the restricted mortality model. Residual confounding related to prior quinolone exposure remains possible. |
| Mortality | CRKP BSI (vs. CSKP BSI) | 0.002 | Yes | Retained as the primary resistance-phenotype variable of clinical interest to assess its adjusted association with mortality after accounting for age and early illness severity. Retention was based on clinical relevance and the study objective, not on final statistical significance. |

**Note: Variables listed met the univariable screening criterion (P < 0.05, based on unrounded values) in Table 4 for the CRKP-versus-CSKP comparison or in the overall cohort comparison in Table 6 for mortality. The final covariate sets were not fully prespecified; the selection process is described in the Statistical Analysis section. Listing a variable does not imply that it was entered into a fitted multivariable model. Clinical prioritization and overlap between composite and component variables were not treated as evidence of statistical multicollinearity.**

**Abbreviations: BSI, bloodstream infection; CRKP, carbapenem-resistant *Klebsiella pneumoniae*; CSKP, carbapenem-susceptible *Klebsiella pneumoniae*; ICU, intensive care unit; BLBLIs, β-lactam/β-lactamase inhibitor combinations.**
